# A novel *adenylate isopentenyltransferase 5* regulates shoot branching via the ATTTA motif in *Camellia sinensis*

**DOI:** 10.1186/s12870-021-03254-5

**Published:** 2021-11-09

**Authors:** Liping Zhang, Menghan Li, Peng Yan, Jianyu Fu, Lan Zhang, Xin Li, Wenyan Han

**Affiliations:** Key Laboratory of Tea Quality and Safety Control, Ministry of Agriculture, Tea Research Institute, Chinese Academy of Agricultural Sciences, No. 9 Meiling South Road, Xihu District, Hangzhou, 310008 Zhejiang China

**Keywords:** Adenylate isopentenyltransferase, ATTTA motif, Alternative splicing (AS) variant, Axillary bud, Cytokinins (CTKs), Tea plant

## Abstract

**Background:**

Shoot branching is one of the important agronomic traits affecting yields and quality of tea plant (*Camellia sinensis*). Cytokinins (CTKs) play critical roles in regulating shoot branching. However, whether and how differently alternative splicing (AS) variant of CTKs-related genes can influence shoot branching of tea plant is still not fully elucidated.

**Results:**

In this study, five AS variants of CTK biosynthetic gene *adenylate isopentenyltransferase* (*CsA-IPT5*) with different 3′ untranslated region (3ˊ UTR) and 5ˊ UTR from tea plant were cloned and investigated for their regulatory effects. Transient expression assays showed that there were significant negative correlations between CsA-IPT5 protein expression, mRNA expression of *CsA-IPT5* AS variants and the number of ATTTA motifs, respectively. Shoot branching processes induced by exogenous 6-BA or pruning were studied, where *CsA-IPT5* was demonstrated to regulate protein synthesis of CsA-IPT5, as well as the biosynthesis of *trans*-zeatin (*t*Z)- and isopentenyladenine (iP)-CTKs, through transcriptionally changing ratios of its five AS variants in these processes. Furthermore, the 3′ UTR AS variant 2 (3AS2) might act as the predominant AS transcript.

**Conclusions:**

Together, our results indicate that 3AS2 of the *CsA-IPT5* gene is potential in regulating shoot branching of tea plant and provides a gene resource for improving the plant-type of woody plants.

**Supplementary Information:**

The online version contains supplementary material available at 10.1186/s12870-021-03254-5.

## Background

Shoot branching determines the aboveground architecture of a plant and lateral branches generally arise from the outgrowth of axillary buds, which is controlled by a complex interaction of phytohormones and environmental conditions [1]. The perennial tea plant (*Camellia sinensis*) is an economically important woody crop, with their bud leaves from lateral branches collected for tea and food production [2, 3]. In virture of such economic value, understanding the molecular regulatory mechanism of shoot branching in tea plant is essential [1].

Cytokinins (CTKs) are primary hormones constituting the various endogenous CTKs in plants [4, 5]. Though mainly biosynthesized in roots, CTKs can also be locally biosynthesized to facilitate shoot [branching, in stem nodes, axillary buds and stems etc. [4, 6]. Isopentenyltransferase (IPT) catalyzes the first and rate-limiting step of CTK biosynthesis [7, 8]. The isopentenyladenine (iP)-type CTKs biosynthesized in the stem node of tomato (*Solanum lycopersicum*) promote axillary bud sprouting and lateral branch growth [4]. Decapitation significantly induces the increase of *PsIPTs* expression in nodal stem tissues as well as CTK levels in the nodal tissue and axillary buds of pea (*Pisum sativum*) [3]. Adenylate IPT (A-IPT), as one of the two types of IPTs, are likely to be responsible for the bulk of CTK synthesis, including iP- and *trans*-zeatin (*t*Z)-type CTKs [3, 8, 9]. Cook et al. [10] reported that the upregulated expression of *A-IPT* in axillary buds and stem nodes stimulates the outgrowth of axillary buds. Overexpression of the *A-IPT* gene in *Asakurasanshoo* [7], *Chrysanthemum* [1, 8] and *Arabidopsis* [8] resulted in increased branching.

Alternative splicing (AS) post-transcriptionally regulates gene expression, leading to differential mRNA stability and enriched protein diversity [11, 12]. The untranslated regions (UTRs) are generally involved in post-transcriptional regulation, which may affect mRNA stability and translation efficiency [13]. In *Arabidopsis*, 21.6% of AS events occur in UTRs [14]. The mRNA stability of the *Arabidopsis SAUR26* gene has been conferred by polymorphisms in the 3′ UTR region and shown to control gene expression in response to environmental factors [13].

AU-rich elements (AREs) are sequence elements of 50-150 nt that are rich in adenosine and uridine bases and are frequently found in the 3′ UTRs of labile mRNAs [15, 16]. In higher eukaryotes, the AUUUA motif, which are among the best-characterized mRNA-destabilizing determinants, and other AREs could decrease gene expression and inhibit mRNA translation (i.e., protein synthesis), at least partially by enhancing the rate of its deadenylation and subsequent mRNA degradation [16–22]. The ATTTA motif was found to be significantly enriched in transcripts with short half-lives in plants [20, 23, 24]. In the presence of three AUUUA sequences in the 3′ UTR, transcripts of tobacco (*N. tabacum*) *par* gene have shorter lifes [20]. Mutations in the AUUUA motifs increase mRNA stability and enhance mRNA accumulation [17, 24], whereas insertion of AUUUA motifs into a stable mRNA destabilizes the mRNA levels [15, 20]. ATTTA and other AREs-specific stability and deadenylation of mRNA appear to be regulated by development and stress [17, 20–22]. In *Arabidopsis*, modulation of mRNA stability contributes to the clock-regulated expression of *AtGUTs* which contain AUUUA motifs [25]. In mammalian cells, many early-response genes are also regulated by instability of mRNAs containing AUUUA motifs within the 3′ UTR [17].

To date, the molecular mechanism that regulates shoot branching in tea plant has not been elucidated. In this study, alternatively spliced *CsA-IPT5* mRNAs with different 3ˊ UTR and 5ˊ UTR were cloned. The relationships among the expression of *CsA-IPT5* transcripts and CsA-IPT5 protein, as well as CTKs biosynthesis were examined in the shoot branching induced by exogenous 6-BA treatment and pruning, respectively. The results are expected to reveal the molecular mechanism that regulates plant shoot branching by splice variants of *CsA-IPT5* and lay a candidate gene resources for breeding tea varieties.

## Results

### Cloning of *CsA-IPT* gene by 3′ and 5′ rapid amplification of cDNA ends (RACE)

The first-round 3′ RACE PCR at different temperatures all resulted in three gel bands, with approximate sizes of 0.9, 1.0, and 1.1 kb, respectively (Figure S1A, lane A). The second-round 3′ RACE PCR obtained gel bands with similar sizes of 0.8, 0.9, and 1.0 kb, respectively (Figure S1A, lane B). In the 5′ RACE, several gel bands were amplified at different temperatures in the first-round PCR (Figure S1B, lane A). In the second-round PCR, there was one amplified gel band at six different temperatures, approximately at 600 bp (Figure S1B, lane B). The primers used are shown in Table S1 and S2.

### Sequencing analysis and multiple sequence alignment of the full-length *CsA-IPT* cDNA and CsA-IPT5 protein

**Fig. 1:**
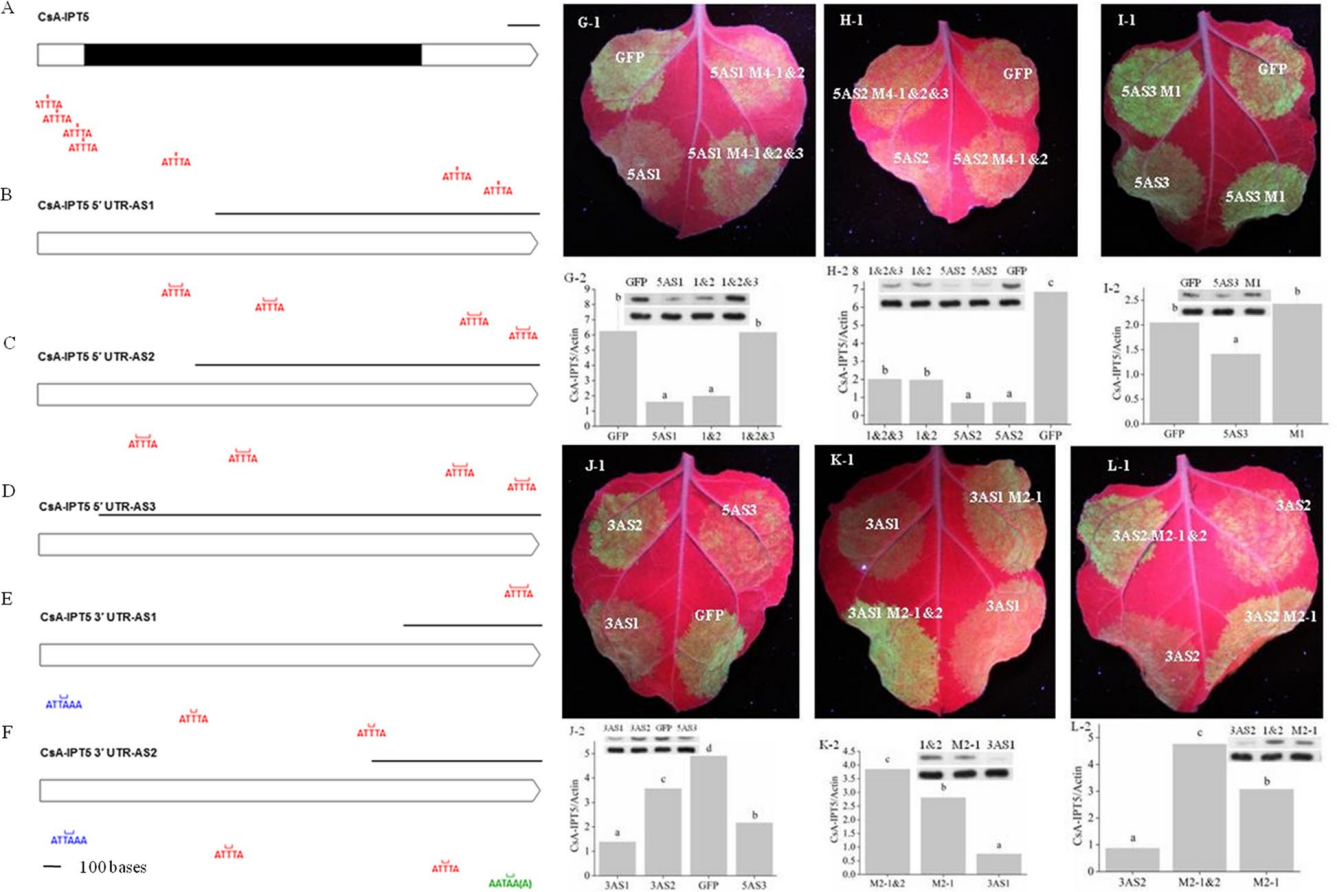
Schematic diagram of the *CsA-IPT5* transcripts, GFP fluorescence and qPCR detection of GFP. **A-F** Schematic diagram of the full-length *CsA-IPT5* cDNA (**A**) and five *CsA-IPT5* splice variants (**B-F**). Black rectangles represent CDS; Hollow rectangles with arrows represent UTR; a line segment represents 100 bases. The ATTTA motif and poly(A) signal sequence has been labeled with different colors, respectively. **G-L** GFP fluorescence and protein expression of GFP. Data are means ±standard deviation (SD) (*n* = 6). In each figure, letters indicate significant differences among the GFP constructs (*P <* 0.05, Duncan’s multiple range test)

Based on the result of sequencing, the 5ˊ UTR and 3ˊ UTR with poly (A) were 145 bp (1-145 bp) and 361 bp (1187-1547 bp) in length, respectively. The full-length cDNA sequence of *CsA-IPT* was 1547 bp in length, and the ORF was 1041 bp (146-1186 bp) responsible for 346 deduced amino acids. There are seven repeats of destabilizing ATTTA motifs in the full-length cDNA sequences of *CsA-IPT*, with four in the 5′ UTR, one in the ORF region, and two in the 3′ UTR (Figure S2). The physical positions of the ATTTA motifs are shown in Fig. 1A.

The sequence homology between the cloned *CsA-IPT* ORF and TEA025674.1 (TPIA, cv. Shuchazao, http://tpia.teaplant.org/index.html?tdsourcetag=s_pcqq_aiomsg) is 99.42%. There is a lack of 67 bases in the cloned *CsA-IPT* compared with the 5′ UTR of XP_028094976.1 (NCBI, https://www.ncbi.nlm.nih.gov/). There are both additional 115 bases in the final 3′ UTR of the cloned *CsA-IPT* compared with that in the 3′ UTR of XP_028094976.1 and XP_028094977.1 (NCBI, https://www.ncbi.nlm.nih.gov/), respectively (Figure S3A, B). In addition, there is a lack of 186 nucleotides in the ORF sequence of the cloned *CsA-IPT* compared with that in the end of the CDS sequence of GWHPACFB026317 (BIG database of cv. LJ43, https://bigd.big.ac.cn/search/?dbId=gwh&q=GWHACFB00000000), and the CDS sequence of GWHPACFB026317 was not obtained by PCR amplification in this study. The phylogenetic tree was constructed using the amino acid sequences of 15 A-IPT protein in different plant species. Sequence alignment analysis showed that the cloned *CsA-IPT* had orthologs in other plant species and belonged to the plant *A-IPT5* gene family. Thus the cloned *CsA-IPT* was termed as *CsA-IPT5* (Figure S3C).

### Multiple sequence alignment of the *CsA-IPT5* AS variants and subcellular localization of CsA-IPT5 protein

**Fig. 2:**
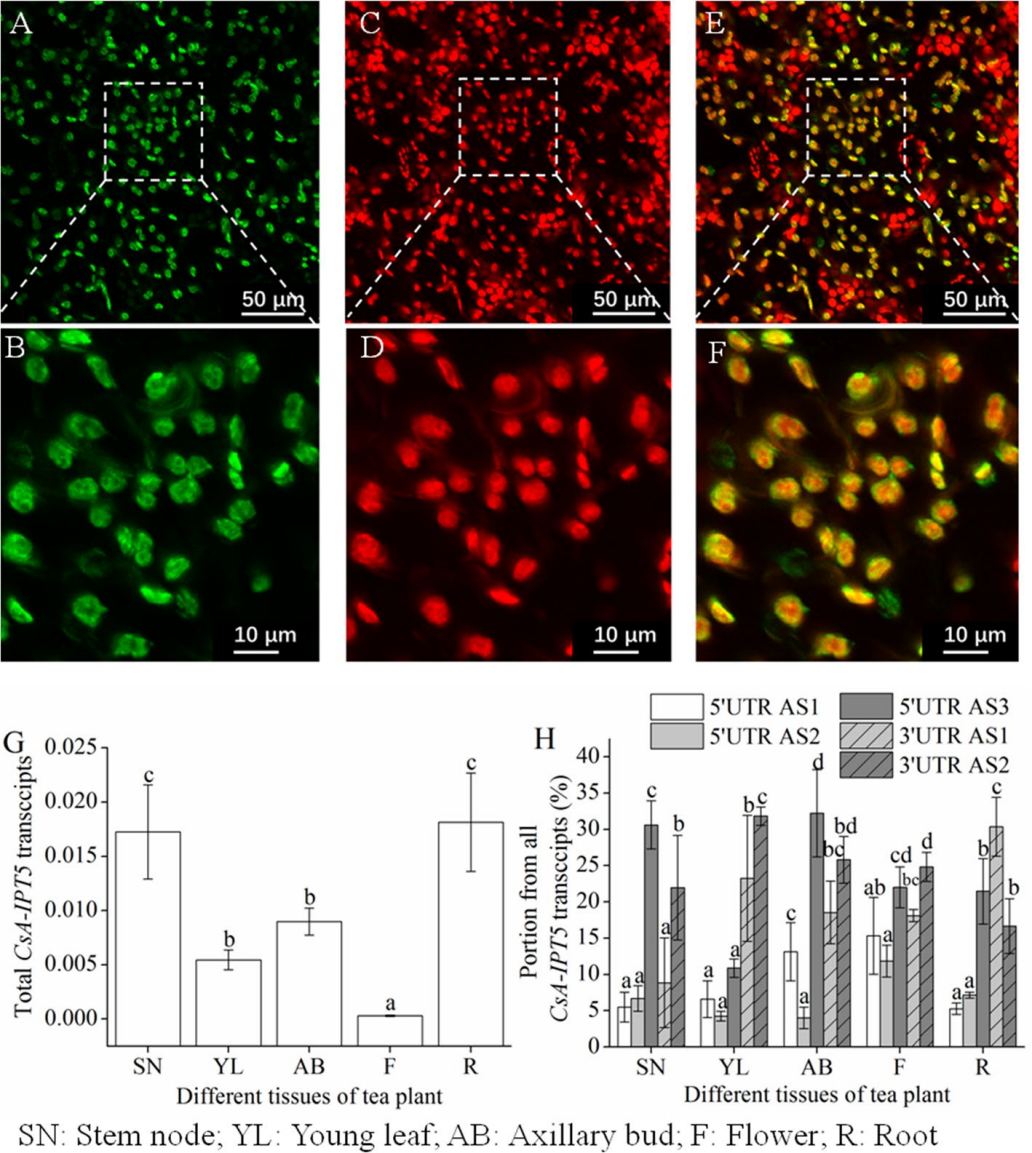
Subcellular localization of CsA-IPT5-GFP and expression profiles of *CsA-IPT5* transcripts in the different tissues of tea plant. **A** and **B** (magnification of **A**), CsA-IPT5::GFP fluorescence (green); **C** and **D** (magnification of **C**), chloroplast autofluorescence (red); **E** and **F** (magnification of **E**), merged (yellow). **G**, **H** Expression profiles of *CsA-IPT5* transcripts in the different tissues of tea plant. AS patterns were expressed as ratios of the indicated splice variants to the total transcripts, and the data are mean ± SD (*n* = 6). Letters indicate significant differences of total *CsA-IPT5* among tissues (**G**) and among splice variant ratios in each tissue (**H**) (*P <* 0.05, Duncan’s multiple range test)

Both four AS variants were found in the 5′ UTR and 3′ UTR of *CsA-IPT5*, respectively. Variation of ATTTA motifs in the UTRs of 5′ UTR AS variant (5AS) and 3′ UTR AS variant (3AS) mRNAs are shown in Fig. 1B-F and Figure S4. Specifically, there are four ATTTA motifs in the 5′ UTR of 5AS1 and 5AS2 mRNA, while there is both only one ATTTA motif in the 5′ UTR of 5AS3 and 5AS4 mRNA, respectively. Likewise, there are two ATTTA motifs in the 3′ UTR of 3AS1 and 3AS2 mRNA, while there is both only one ATTTA motif in the 3AS3 and 3AS4 mRNA, respectively. Furthermore, no change was found in the start or stop codon of *CsA-IPT5*, although AS was detected in the 5′ RACE and 3′ RACE (Figure S4), indicating the splicing variants will be translated into the same protein product-CsA-IPT5. The result of subcellular localization showed that the CsA-IPT5 protein located in the chloroplasts (Fig. 2A-F).

### Effect of the ATTTA motifs on CsA-IPT5 protein expression and transcriptional activity of *CsA-IPT5* transcripts in *N. benthamiana* leaves by multiple mutation

Both 5AS1 and 5AS2 contain four ATTTA motifs (Fig. 1B, C; Figure S5A-1, B-1). For these two, we created mutants of M4-1&2 and M4-1&2&3, with the first two and the first three ATTTA motifs altered to GCCCG motifs, respectively (M represents mutant) (Figure S5A, B). The result showed that the fluorescence, protein expression (Fig. 1G-J) and mRNA expression (Figure S6A-D) in the constructs were all lower than those in the relative GFP controls, except for 5AS1 M4-1&2&3 protein expression and 5AS3 M1 protein expression. Fluorescence was both greater from the GFP-5AS1 M4-1&2 and GFP-5AS1 M4-1&2&3 clones than that from GFP-5AS1. The protein expression of GFP-5AS1 M4-1&2&3 was significantly greater than that of GFP-5AS1, however, there were no significant difference between that of GFP-5AS1 M4-1&2 and GFP-5AS1. Results of qPCR showed that mRNA levels of GFP-5AS1 M4-1&2 and GFP-5AS1 M4-1&2&3 were about four times and 4.3 times, respectively, compared with GFP-5AS1. Moreover, the fluorescence, protein expression (Fig. 1G-1, 2) and mRNA expression (Figure S6A) of GFP-5AS1 M4-1&2&3 was all greater than that from GFP-5AS1 M4-1&2. On the other hand, fluorescence was both greater from the GFP-5AS2 M4-1&2 and GFP-5AS2 M4-1&2&3 clones than that from GFP-5AS2 (Fig. 1H-1). The mRNA levels of GFP-5AS2 M4-1&2 and GFP-5AS2 M4-1&2&3 were both about twice of that resulting from GFP-5AS2, while there was no significant diffenence between that from GFP-5AS2 M4-1&2 and GFP-5AS2 M4-1&2&3 (Figure S6B). Moreover, the protein expression showed exactly the same pattern with the above gene expression (Fig. 1H-2). The sequence of 5AS3 contains only one ATTTA motif which was mutated into GCCCG to create the 5AS3 M1 mutant (Figure S5C; Fig. 1D). Fluorescence, protein expression (Fig. 1I-1, -2) and mRNA expression (Figure S6C) were all significantly greater than that from the GFP-5AS3 M1 than from GFP-5AS3.

The ATTTA motif both occurs twice in 3AS1 and 3AS2, respectively. In addition, 3AS1 and 3AS2 have one and two ATTAA(A) polyA signal sequence(s), respectively (Figure S5D, E; Fig. 1E, F). The fluorescence, protein expression (Fig. 1J-1, J-2), and mRNA expression (Figure S6D) in 3AS2 were significantly greater than that in 5AS3, and that in 3AS2 were significantly greater than that in 3AS1. The fluorescence, protein expression (Fig. 1K-1, -2) and mRNA levels (Figure S6E) in 3AS1 M2-1&2 was significant greater than that in 3AS1 M2-1, and that in these two appeared to be all greater than that in the GFP-3AS1. The fluorescence, protein expression (Fig. 1L-1, -2) and mRNA levels (Figure S6F) in the 3AS2 M2-1 and 3AS2 M2-1&2 clones were both greater than that in the GFP-3AS2, and that in GFP-3AS2 M2-1&2 was greater than that in GFP-3AS2 M2-1.

### Designing and verification of the qPCR primers for *CsA-IPT5* splice variants

The primers for amplifying different AS transcripts of *CsA-IPT5* were designed. Due to the presence of ASs both in the 5′ and 3′ UTRs, the qPCR primers could not be designed to detect the expression of the full-length *CsA-IPT5* transcript. The corresponding qPCR primers can not be designed due to the small difference in the sequence length between 5AS3 and other splice variants and the higher AT contents in the diversity sequences. In addition, as the sequences of 5AS4 all coincide with those of the other splice variants, the amplified products of the primer pair 5AS4F + 5ASR is the total expression of all the splice variants. Therefore, for the four 5′ UTR-AS and four 3′ UTR-AS obtained by RACE, the designed primers could only be used for distinguishing the expression levels of 5AS1, 5AS2, 5AS3, 3AS1, 3AS2 and the total *CsA-IPT5* transcript (Table S3).

The band size of the primers, including 5AS4F + 5ASR (201 bp, lane 4), 3ASF + 3AS4R (199 bp, lane 5), 3ASF + 3AS2R (304 bp, lane 6), 3ASF + 3AS1R (427 bp, lane 7), 3ASF2 + 3AS1R (328 bp, lane 8), and 3ASF2 + 3AS2R (205 bp, lane 9), all correspond to the expected size. As the primers 3ASF + 3AS4R (lane 5), 3ASF + 3AS1R (lane 8) and 3ASF + 3AS2R (lane 9) have higher amplification efficiency compared to the primers 5AS4F + 5ASR (lane 4), 3ASF2 + 3AS1R (lane 7) and 3ASF2 + 3AS2R (lane 6), respectively, thus these three primer pairs are recommended for detcting the expression of the total transcript, 3AS1 and 3AS2 of *CsA-IPT5*, respectively. CsGAPDH F + CsGAPDH R1 was used for detecting the expression of housekeeping gene *GAPDH* (Figure S7A).

The temperature gradient amplification of different 5′ UTR AS was carried out. The primer pairs 5AS1F + 5ASR, 5AS2F + 5ASR and 5AS3F + 5ASR were used to amplify 5AS1, 5AS2 and 5AS3, respectively. Lines A1 ~ A6, B1 ~ B6 and C1 ~ C6 correspond to the amplified results of primer pairs 5AS1F + 5ASR (247 bp), 5AS2F + 5ASR (247 bp) and 5AS3F + 5ASR (249 bp) under different temperature, respectively. The primer pairs 5AS1F + 5ASR and 5AS2F + 5ASR both could amplify the objective band, respectively. Moreover, the primer pairs both have higher amplification efficiency under 54 °C and 55 °C compared with other temperature (Figure S7B).

### The expression of *CsA-IPT5* transcripts in the different tissues of tea plant

The expression patterns of *CsA-IPT5* transcripts in the different tissues of tea plant were detected. The results showed that the *CsA-IPT5* transcripts expressed in all the tested tissues. The total *CsA-IPT5* transcripts in the stem node and root were the highest, followed by that in the young leaf and axillary bud. In the stem node, the 5AS3 expression/total expression (5AS3 ratio) and 3AS2 expression/total expression (3AS2 ratio) was significantly higher than the other three splice variants; in the young leaf, the 3AS1 ratio and 3AS2 ratio both were significantly higher than the other three splice variants; in the axillary bud, except for 5AS2, the other four splice variants all have relatively high expression levels, and the 5AS3 ratio was the highest among them; in the flower, all five splice variants have a relatively high expression level, and the 5AS1 ratio and 5AS2 ratio was relatively low compared with the three other splice variants; in the root, 5AS3, 3AS1 and 3AS2 have relatively high expression levels compared with the three other splice variants (Fig. 2G, H). Together, among the five splice variants, 5AS3 had the highest ratio in the stem node and axillary buds, while 3AS2 showed the highest ratio in young leaf and flower, and 3AS1 appeared to be the highest in roots. Furthermore, among the five splice variants of *CsA-IPT5*, 3AS2 all had relatively high ratio in the five tested tissues.

### Involvement of *CsA-IPT5* transcripts in the shoot branching induced by exogenous 6-BA

**Fig. 3:**
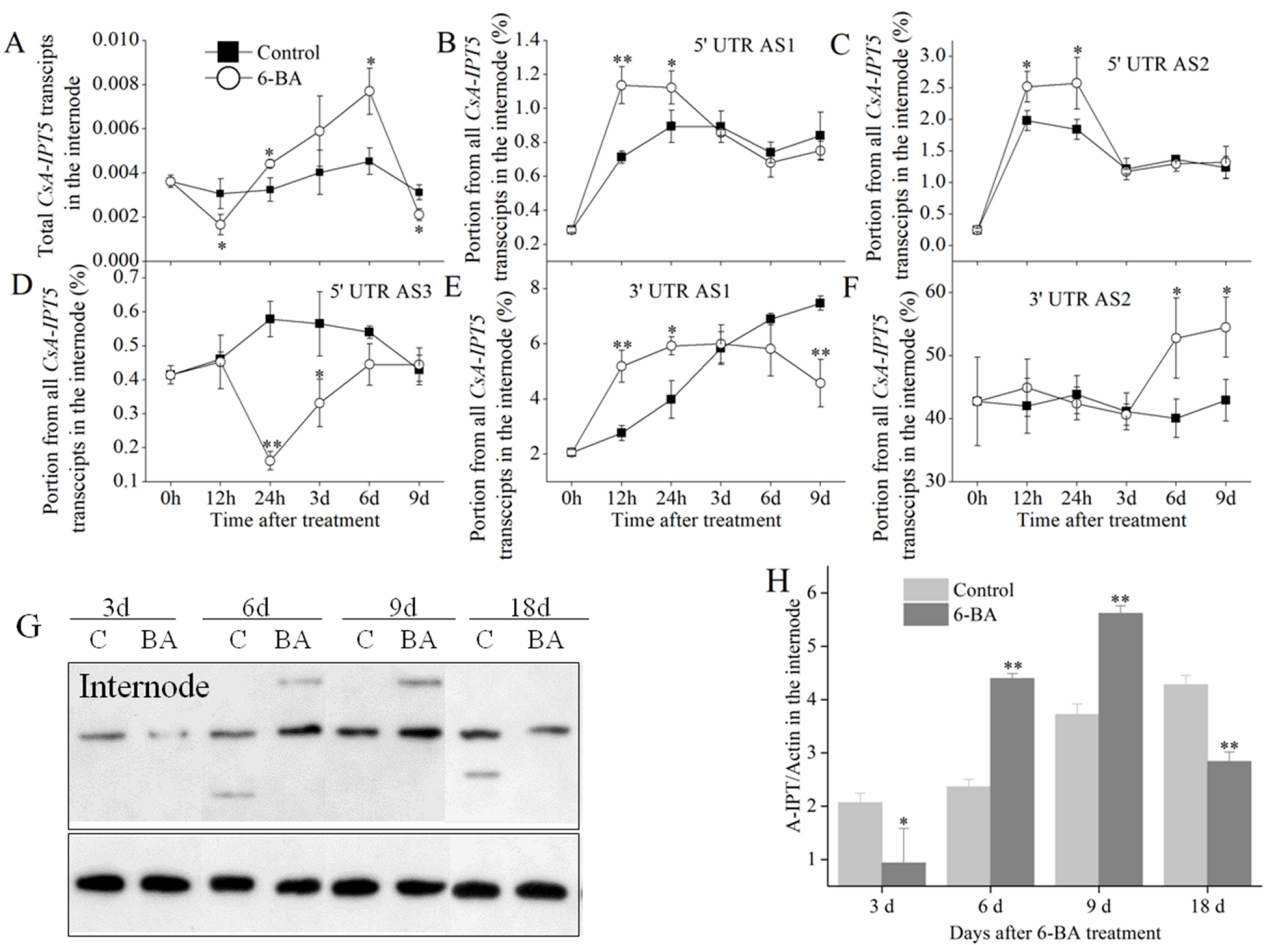
Expression of *CsA-IPT5* transcripts and CsA-IPT5 protein in the internode induced by 6-BA application. **A** Expression of total *CsA-IPT5* transcripts in the internode at different time points induced by 6-BA. **B-F** The ratios of *CsA-IPT5* splice variants in the internode at different time points induced by 6-BA. Western blot (**G**) and ‘CsA-IPT5/Actin’ (**H**) which showed CsA-IPT5 protein expression in the internode at 3 d, 6 d, 9 d and 18 d after 6-BA treatment. Data are means ±SD (*n* = 3 or 6). For figures **A-F**, asterisks indicate significant differences in each index between control and 6-BA treatment at each time point; for figure **H**, asterisks indicate significant differences in A-IPT/Actin between control and 6-BA treatment at each time point (**P < 0.01; Student’s *t*-test)

In the internode, 6-BA induced the expression of total *CsA-IPT5* transcripts, 5AS1, 5AS2, 3AS1, and 3AS2 compared with the relevant controls. Specially, in the internode, the total *CsA-IPT5* transcripts induced by 6-BA were both significantly higher at 24 h and 6 d compared with the control. The 5AS1, 5AS2 and 3AS1 ratio induced by 6-BA was all significantly higher than that in the relative control at 12 h and 24 h. The 3AS2 ratio induced by 6-BA was significantly higher than that in the control at 6 d and 9 d. However, 6-BA did not increase the 5AS3 ratio compared with the control (Fig. 3; Table S4).

**Fig. 4:**
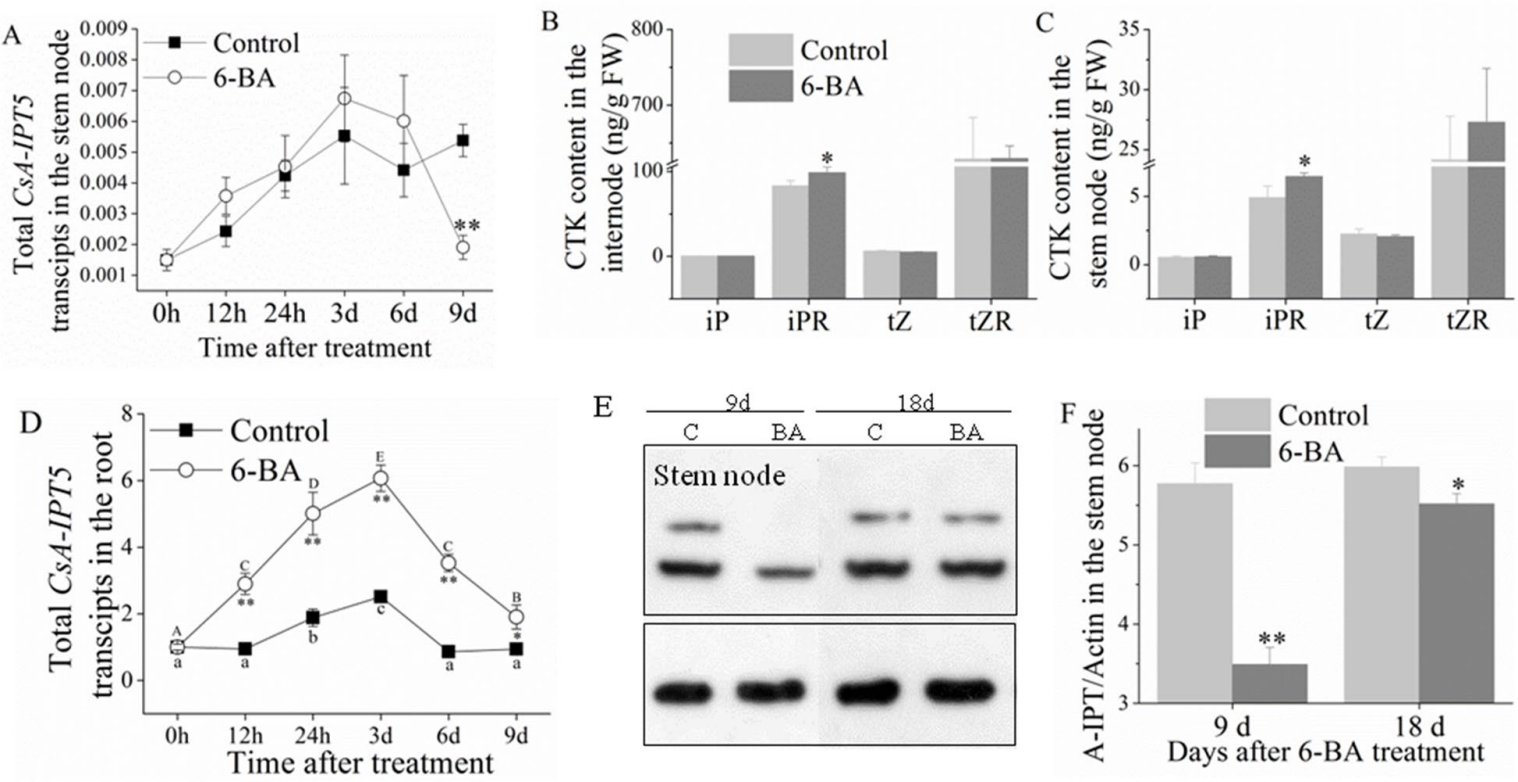
Expression of total *CsA-IPT5* transcripts in the stem node and root, CTKs contents in the internode and stem node, and expression of CsA-IPT5 protein in the stem node induced by 6-BA application. **A**,**D** Expression of total *CsA-IPT5* transcripts in the stem node (**A**) and root (**D**) at different time points induced by 6-BA. **B**, **C** CTK contents in the internode (**B**) and stem node (**C**) induced by 6-BA. Western blot (**E**) and ‘CsA-IPT5/Actin’ (**F**) which showed CsA-IPT5 protein expression in the stem node at 9 d and 18 d after 6-BA treatment. Data are means ±SD (*n* = 3 or 6). For figures **A** and **D**, asterisks indicate significant differences in the expression of total *CsA-IPT5* transcripts between control and 6-BA treatment at each time point; for figures **B** and **C**, asterisks indicate significant differences in each index between control and 6-BA treatment; for figure **F**, asterisks indicate significant differences in ‘CsA-IPT5/Actin’ between control and 6-BA treatment at each time point (**P < 0.01; Student’s *t*-test)

In the stem node, the total *CsA-IPT5* transcripts both showed a trend of first increasing and then decreasing in the control and 6-BA treatment, respectively, and 6-BA did not increase the expression of total *CsA-IPT5* transcripts compared with the control (Fig. 4A). In the root, the expression of the total *CsA-IPT5* transcripts in the 6-BA treatment was all significantly higher than that in the control at all the test time points (Fig. 4D). The iPR content both increased in the internode and stem node when treated with 6-BA (Fig. 4B, C). The protein expression of CsA-IPT5 in the internode was induced by 6-BA at 6 d and 9 d compared with the control (Fig. 3G, H), however, it did not induce the increase of CsA-IPT5 protein expression in the stem node (Fig. 4E, F).

### Involvement of *CsA-IPT5* transcripts in regulating axillary bud development induced by pruning

**Fig. 5:**
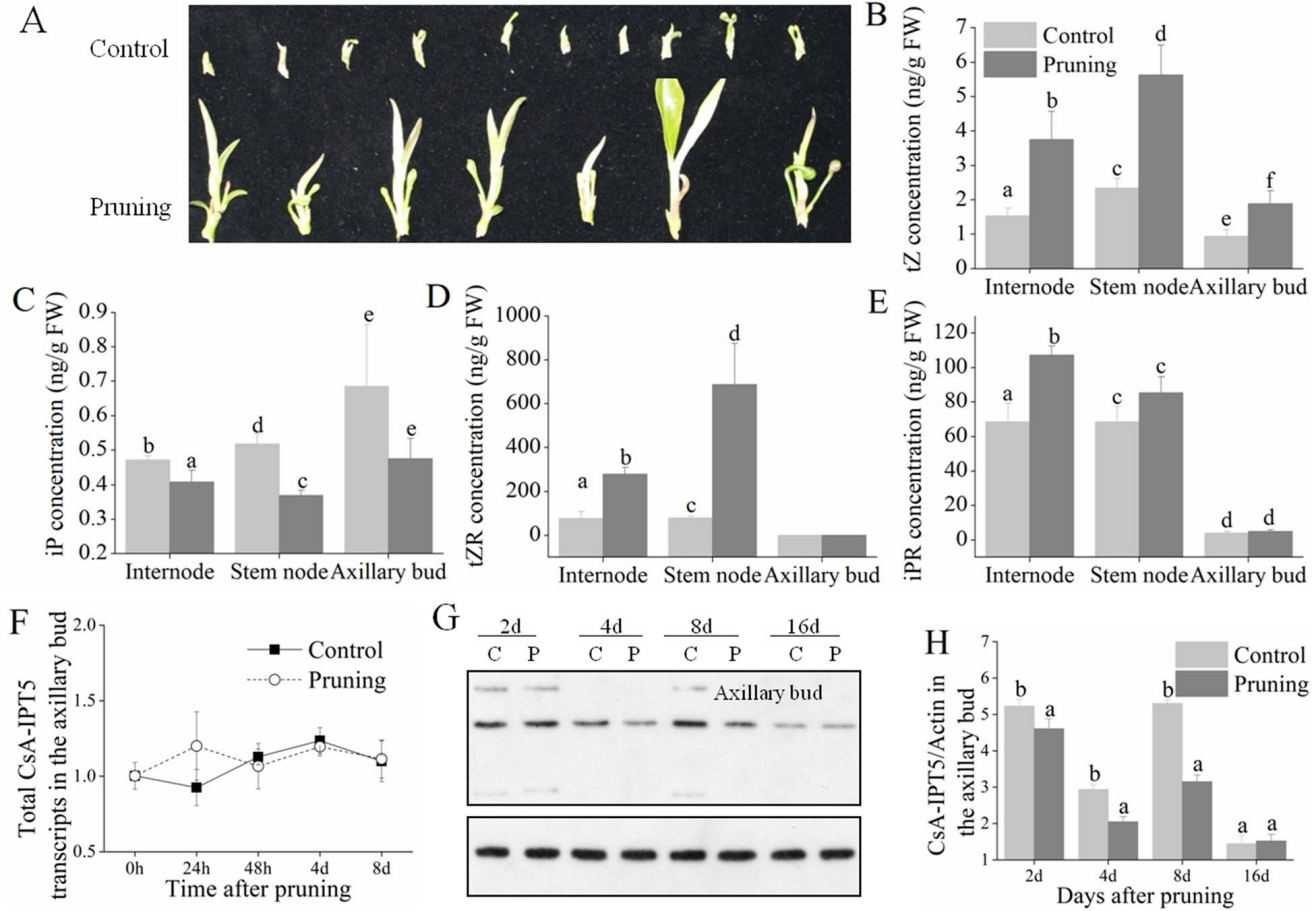
The axillary bud growth vigor, CTK contents in the three tissues, and the expression of the total *CsA-IPT5* transcripts and CsA-IPT5 protein in the axillary bud of the first node below the cuttings induced by the second pruning. **A** The growth vigor of the axillary bud at 16 d after pruning. **B-E** Contents of *t*Z- and iP-type CTKs in the different tissues at 8 d after pruning. Data are means ±SD (*n* = 3). For each figure, letters indicate significant differences in the CTK concentration between control and pruning in each tissue (*P <* 0.05, Duncan’s multiple range test). **F** Expression of the total *CsA-IPT5* transcripts in the axillary bud at different time points after pruning. **G**, **H** Western blot (**G**) and ‘CsA-IPT5/Actin’ (H) which showed the CsA-IPT5 protein expression levels in the axillary bud at different time points after pruning. Data are means ±SD (*n* = 3). Letters indicate significant differences of CsA-IPT5/Actin between control and pruning at each time point (*P <* 0.05, Duncan’s multiple range test)

The morphology and size of the axillary buds at 16 d after pruning are shown in Fig. 5A. Compared with the control, pruning induced the accumulation of *t*Z, *t*Z riboside (*t*ZR) and iP riboside (iPR) in the internodes, *t*Z and *t*ZR in the stem node, as well as *t*Z in the axillary buds, respectively, at 8 d after pruning (Fig. 5B-E). In the axillary bud, there was no change in the overall transcripts of *CsA-IPT5* either in the control or in the pruning groups, and pruning did not induce the CsA-IPT5 protein expression in the axillary buds compared with the control (Fig. 5F-H).

**Fig. 6:**
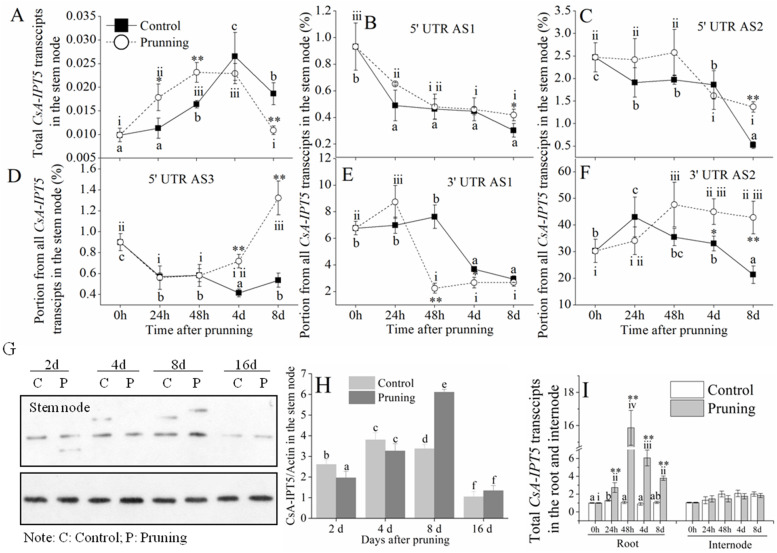
Expression of *CsA-IPT5* transcripts and CsA-IPT5 protein in the stem node, the expression of total *CsA-IPT5* transcripts in the internode of the first node below the cuttings and root at different time points after the second pruning. **A**-**H** The expression of total *CsA-IPT5* transcripts (**A**), *CsA-IPT5* splice variants (**B-F**), and CsA-IPT5 protein (**G**, **H**) in the stem node after the pruning. **I** The expression of total *CsA-IPT5* transcripts in the root and internode at different time points after pruning. Data are means ±SD (*n* = 6 or 3). For each figure, asterisks indicate significant differences in each index between control and pruning at each time point (**P < 0.01; Student’s *t*-test). Except for figure **H**, letters indicate significant differences of gene expression for each treatment among different time points (*P <* 0.05, Duncan’s multiple range test) (**A-F**). For figure **H**, letters indicate significant differences of CsA-IPT5/Actin between control and pruning at each time point (*P <* 0.05, Duncan’s multiple range test)

In the stem node, the total *CsA-IPT5* transcripts in the control and pruning groups both increased at first and then decreased from 0 h to 8 d, and that in the the pruning groups were significantly higher than that in the control at 24 h and 48 h (Fig. 6A). The 5AS3 ratio in control showed a trend of gradual decreasing, whereas it showed a trend of first decreasing and then increasing after pruning in the stem node (Fig. 6D). The 3AS2 ratio in control of the stem node showed a trend of first increasing and then decreasing, whereas it showed a trend of gradual increasing in the pruning treatment (Fig. 6F). Thus, compared with the relevant control, the pruning treatment induced the expression of 5AS3 and 3AS2 respectively, in the stem node. The ratios of 5AS1, 5AS2 and 3AS1 all did not increase both in the control and pruning treatment over time (Fig. 6B, C, E). Furthermore, compared with the control, pruning induced the protein expression of CsA-IPT5 in the stem node at 8 d after pruning (Fig. 6G, H). Compared with the control, the expression of total *CsA-IPT5* transcripts was induced by pruning in the roots but not in the internode at all the tested time points (Fig. 6I).

## Discussion

Abundance of leaf buds during shoot branching is indespensible for achieving high yield and quality of tea plant, thus artificially regulating the development of the lateral branches is necessary for tea production as its obvious apical dominance [2]. A-IPTs play essential roles in the synthesis of iP- and *t*Z-type CTKs [9, 26]. Up to now, the function of plant *A-IPTs* has been much studied, whereas little has been done to characterize the splice variants of plant *A-IPT* transcripts. In the present study, clones of alternatively spliced *CsA-IPT5* mRNAs of tea plant with different 3ˊ UTR and 5ˊ UTR were identified.

Some sequences in the UTRs, such as AU-rich elements (ARE), could modulate mRNA stability and thus regulate the transcript accumulation and translation efficiency of the mRNA [19, 27]. Most of the reported sequences involved in mRNA stabilization reside within the 3′ UTR [28], meanwhile, Chen et al. [29] reported that the ATTTA-containing core domain in the 5′ UTR of human cytokine mRNA functions as a potent destabilizing element. The 5′ UTR can also be involved in modulating mRNA stability during the development and stress response of green plants [30–33]. In this study, there are one to four ATTTA motif(s) in the five 5′ UTR and 3′ UTR splice variants which were investigated for their regulatory effects.

In the current study, the biggest difference among the splice variants is in the repeats of the unstable ATTTA motifs. Our transient expression and mutation test showed differences in the repeats of the ATTTA motifs, varying from zero to four, in the fused GFP. The resuts showed that except for two mutants of 5AS2 (i.e., 5AS2 M4-1&2 and 5AS2 M4-1&2&3), there were all negative correlations between the transcriptional levels and the number of ATTTA motifs among the *CsA-IPT5* splice variant and its relevant mutants. Furthermore, except for 5AS1 and 5AS1 M4-1&2, as well as 5AS2 M4-1&2 and 5AS2 M4-1&2&3, there were all negative correlations between the CsA-IPT5 protein expression and the number of ATTTA motifs among the AS variant and its relevant mutants. This seemed to be consistent with a number of previous studies which consider that transcripts containing a larger amount of ATTTA motifs and other ARE are more rapidly degraded than those containing a smaller amount of these motifs [17, 34]. In addition, this study showed that there were both no significant differences about the protein expression and transcriptional levels of fused GFP between that in the 5AS2 M4-1&2 and 5AS2 M4-1&2&3, which have two and one ATTTA motif(s) respectively, and this is consistent with an existing report that the accumulate rates of two alternative spliced mRNAs, which with one and two ATTTA motif(s) respectively, were not distinct in vitro [24].

The poly(A) consensus signal sequence, including the AATAA(A) motif and the less frequently found ATTAAA motif, located in the 3ˊ UTR of the mature mRNA, is essential for poly(A) tail formation during mRNA maturation [35, 36]. He et al. [24] reported that the ATTAAA sequence and AATAA sequence in the 3ˊ UTR both upregulated plant gene expression. Here, there are two ATTTA motifs and two ATTAA(A) motifs in the 3AS2, and there is one ATTTA motif and no poly(A) signal sequence in the 5AS3. Thus, compared with 5AS3, the significantly greater fluorescence and mRNA levels of 3AS2 may be attributed to the upregulated gene expression confered by the two AATAA(A) motifs. Likewise, compared with 3AS1, the higher expression levels of 3AS2 may be also attributed to the one more AATAA(A) motif in the 3AS2. Therefore, it is likely that the poly(A) signal sequence might be responsible for the higher transient expression levels of *CsA-IPT5* splice variants, and the final transient expression level of *CsA-IPT5* transcripts may be the result of the interplay of the two regulatory mechanisms that can act in opposing directions.

Studies in multiple plants have indicated that each organ or tissue has its own specific AS transcripts, and tissue specific AS may have potential functions [37]. This study showed that the *CsA-IPT5* splice variants are expressed in all the tested tissues, including the stem node, young leaf, axillary bud, flower, and root, and their spatial expression patterns revealed a tissue preference and differences in expression levels. Furthermore, among the five splice variants of *CsA-IPT5*, 3AS2 all had relatively high ratio in the five tested tissues.

**Fig. 7:**
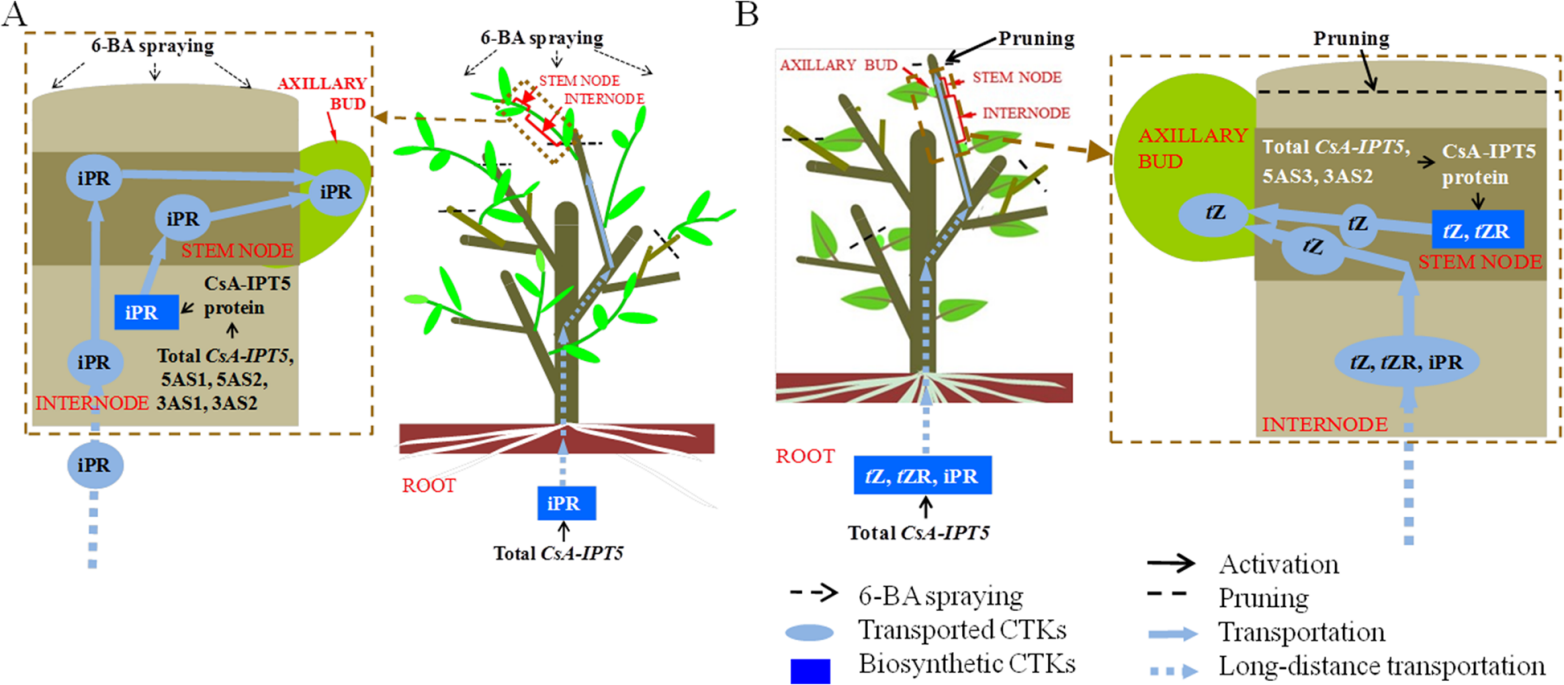
Proposed models of *CsA-IPT5* transcripts in the regulation of shoot branching in tea plant. **A** A proposed model of *CsA-IPT5* transcripts in the regulation of shoot branching induced by exogenous 6-BA application in tea plant. **B** A proposed model of *CsA-IPT5* transcripts in the regulation of shoot branching induced by the second pruning in tea plant. Each shoot branching process regulated by *CsA-IPT5* transcripts are included in a single rectangle composed by brown dashed lines, respectively. Black dashed arrows represent exogenous 6-BA spraying. Black dashed lines represent the second pruning. Light blue ellipses represent the transported CTKs. Blue rectangles represent locally biosynthetic CTKs. Black arrows represent activation. Light blue solid arrows represent transportation, whereas light blue dashed arrows represent long-distance transportation.

Shoot branching of plants includes two stages, the formation and the subsequent elongation growth and development of axillary buds. 6-BA is a kind of synthetic cytokinin inside the plant. Our previous studies showed that 6-BA application increased the number of lateral buds 1 month after treatment, thus promoted productive lateral branch formation of tea plant [38]. The current study showed strong correlations among the increased expression of total *CsA-IPT5* transcripts, 5AS1, 5AS2, 3AS1, 3AS2, increased protein expression of CsA-IPT5, and increased iPR concentrations in the internode induced by 6-BA. Nevertheless, the results indicated 6-BA did not induce the expression of *CsA-IPT5* transcripts and CsA-IPT5 protein in the stem node compared with the control. Thus, 6-BA may induce iPR biosynthesis in the internode, meanwhile, the increased iPR concentration detected in the internode might also be transported from root, as 6-BA increased the expression of total *CsA-IPT5* in the root compared with the control. Thus, the increased iPR concentration detected in the stem node might be transported from the internode and root. Together, it can be speculated that after 6-BA treatment, the iPR biosynthsized in the internode and root was transported to the axillary bud and promote its development (Fig. 7a). Furthermore, the 5AS1, 5AS2, 3AS1 and 3AS2 all played key roles in the shoot branching induced by 6-BA.

Pruning is an essential agronomic practice in tea cultivation [39, 40]. In this study, the increased expression of total *CsA-IPT5* transcripts, 5AS3 and 3AS2, increased protein expression of CsA-IPT5, as well as increased concentrations of *t*Z and *t*ZR in the stem node induced by pruning treatment were shown to be positively correlated. Thus the increased concentrations of *t*Z and *t*ZR detected in the stem node at least partly biosynthsized by itself. Furthermore, pruning induced the expression of total *CsA-IPT5* transcripts in the root but not in the internode. Thus, it can be speculated that the increased concentrations of *t*Z, *t*ZR and iPR in the internode tested here may come from root. However, as pruning did not induce the expression of total *CsA-IPT5* transcripts and CsA-IPT5 protein in the axillary bud, it is possible that the increased *t*Z accumulation in the developing axillary bud is transported from other tissues, including the stem node and root, and promoted the axillary bud developement (Fig. 7b). Furthermore, in the axillary bud development induced by pruning, the 5AS3 and 3AS2 both played key roles.

The regulatory effects of ATTTA motif and other ARE during different developmental stages or under environmental stimui have been frequently reported in higher eukaryotes. For example, optimal metabolic adaptation to oxygen variations relies on ARE-mediated mRNA stability and thus the regulation of gene transcription and enzyme activity in *Drosophila* cells [22]. The splice variants of *high-glucose-regulated* (*HGRG-14*) mRNA, which contains different numbers of ATTTA motifs, is produced and correlates well with HGRG-14 protein levels under different glucose conditions in human mesangial cells [19]. As discussed above, it is most likely that during shoot branching induced by 6-BA treatment or pruning, the expression patterns of the five UTR splice variants of *CsA-IPT5* were differently regulated in this study. The splice variants regulate the synthesis of CsA-IPT5 protein as well as tZ- and iP- CTKs through changing the ratio of the five mRNAs. Furthermore, the 3AS2 might act as the most important transcript.

## Conclusions

In summary, the present study examined the molecular mechanisms of shoot branching regulated by the AS transcripts of *CsA-IPT5* in tea plant. The five *CsA-IPT5* splice variants might play roles through compensation or competition with each other. Of the five, the 3AS2 of *CsA-IPT5* may be the predominant transcript and act as a key determinant with specific functions in the dynamic equilibrium. This study highlights the underlying AS-based regulatory mechanisms in tea plant shoot branching, and it provides a material (gene) for improving the plant-type of woody plants.

## Methods

### Plant material and growth conditions

Adult tea plants (*C. sinensis* L.) of the widely grown cultivars in China, Longjing 43 (LJ43) and Zhongcha 108 (ZC108), were used in this study. All experiments were conducted in the tea garden of the Tea Research Institute, Chinese Academy of Agricultural Sciences, Hangzhou, Zhejiang province, China. No permissions were necessary to collect such samples.

### Isolation of the full-length cDNA of *CsA-IPT*

In the preliminary experiments, due to the lack of genomic data of tea plant cv. LJ43, we obtained the full-length cDNA of *CsA-IPT* in *C. sinensis* cv. LJ43 using RACE technology. The total RNA was extracted from the tea leaves of cv. LJ43 using the modified CTAB method. The first-strand cDNA for 5′ RACE and 3′ RACE of *CsA-IPT* was synthesized using Tiosbio SUPERSWITCH™ PCR cDNA synthetic kit (ST0091, Baoying Tonghui Biotechnology Co., Ltd., Beijing, China), respectively. Purified PCR products were cloned into the pMD18-T vector (*pEASY-T5 Zero* Cloning Kit, TransGen Biotech, Beijing, China), then transformed into the Trans1-T1 phage resistant chemically competent cell (TransGen Biotech). DNA sequencing was performed by TsingKe (Hangzhou, China). The verified forward primer of real-time quantitative PCR (qPCR) (CsIPT5-F query_L1) and the 3′ adaptor primers (3AP) provided in the RACE kit were used for the 3′ RACE-PCR at first, and then performed by nested PCR using the primers CsIPT5-3R2 and 3AP. The first amplification of the 5′ RACE was performed using the primers CsIPT5-5R1 and 5AP and then performed by nested PCR using the primers CsIPT5-5R2 and 5AP in the kit. The primers and the primer combinations used for RACE PCR are shown in Tables S1 and S2. The gradient program for RACE PCR was: 94 °C for 5 min, followed by 35 or 31 cycles of 94 °C for 30 s, 50-68 °C for 30 s, and 72 °C for 3.5 min, and then a 10 min final extension at 72 °C.

### Bioinformatics analysis of the cloned *CsA-IPT* gene

The *CsA-IPT5* cDNA sequence, deduced amino acid sequence, and open reading frame (ORF) were analyzed online or by the corresponding bioinformatics software. The deduced *CsA-IPT5* and other known plant *A-IPT* genes were aligned with online CLUSTALW (http://www.ebi.ac.uk/Tools/msa/clustalw2).

### Subcellular localization of CsA-IPT5

The primers for amplifying the *CsA-IPT5* cDNA full-length ORF without the stop codon was designed (forward primer: 5′-CgACgACAAgACCgTcaccATGAGAATTTCATTCTCACCT-3′; reverse primer: 5′-GAGGAGAAGAGCCGTCGCCGAGTTGTGGCGGCGACA-3′), and the ORF sequence was fused to the green fluorescence protein (GFP, accession number U87973). The fusion gene was inserted into the plant binary expression vector *pJP186* that contains the GFP, yielding the transformation vector 35S: *A-IPT*-GFP. This vector was used for the transient transformation of *Nicotiana tabacum* leaves. The epidermal cells of *N. tabacum* leaves were observed for subcellular localization of *CsA-IPT5* using a confocal laser scanning microscope (Nikon A1, Japan). GFP fluorescence was excited at 488 nm and detected using a 492-548 nm emission filter. Chlorophyll autofluorescence was excited at 543 nm and detected using a 620-700 nm emission filter.

### Transient expression of *CsA-IPT5* splice variants specific GFP of 5′ and 3′ UTR in transfected *N. benthamiana*

Different repeats of ATTTA elements within the splice variants in 5′ and 3′ UTR of *CsA-IPT5* were mutated to GCCCG by oligonucleotide-directed mutagenesis using the altered sites mutagenesis Kit (Promega, Madison, WI, USA) [27]. The splice variants and the mutated genes, as well as the corresponding sequences, are shown in Figure S5. For agroinfiltration, *Nicotiana benthamiana* plants were maintained in growth chambers at 20-25 °C throughout the assays. *Agrobacterium tumefacient* infiltration was performed as described by He et al. [24]. Bacterial cells were harvested and re-suspended in infiltration buffer to a final OD_600_ = 1.0. In co-infiltration experiments, equal volumes of each suspension were mixed before infiltration. GFP fluorescence was observed under a long-wavelength UV light (Black Ray model B 100A, Ultra-Violet Products Ltd., Cambridge, United Kingdom). The comparable constructs and the pCV1300:GFP control were infiltrated into a single leaf to reduce the background difference of the tobacco leaves.

### Real-time quantitative PCR (qPCR)

Total RNA was isolated using an RNA extraction kit (Tiangen Biotech, Beijing, China) and reverse transcribed using the PrimeScriptTM RT-PCR Kit (TaKaRa, Otsu, Japan), following the manufacturer’s instructions. The qPCR assay was performed on an ABI 7500 Real-Time PCR system (Applied Biosystems, Foster City, CA, USA) using a SYBR Green PCR Master Mix (Takara, Shiga, Japan). The qPCR cycling conditions were as follows: 95 °C for 30 s and 40 cycles of 95 °C for 5 s. *CsGAPDH* was chosen as a stable reference gene for expression normalization. Relative gene expression of the *CsAIPT5* transcripts was calculated by the 2^-△CT^ method, and the expression ratio of each splice variant (i.e., expression of splice variant/total expression) was calculated. All qPCR detection of shoot branching experiments were carried out using six independent biological replicates.

The GFP transcript in transfected tobacco leaves were detected in qPCR using the GFP forward primer (TGTCAGTGGAGAGGGTGAA) and reverse primer (CGTCCTTGAAGAAGATGGTC). The PCR reaction was denatured at 95 °C for 1 min, followed by 45 cycles of 15 s at 95 °C, 20 s at 55 °C, and 20 s at 72 °C. The *N. benthamiana* Ubiquitin C gene (accession Number: AB026056.1) was chosen as the reference gene for qPCR analysis. Relative gene expression of the GFP was calculated by the 2^-△△CT^ method.

### Designing and verification of the qPCR primers for *CsAIPT5* splice variants

Total RNA was extracted using the CTAB method, and genomic DNA was removed. RNA reverse transcription was carried out with HiScript II Q RT SuperMix for qPCR (+gDNA wiper) R223-01 (Vazyme, Nanjing, China). Thirteen primers were designed according to the 5ˊand 3ˊ UTRs of the *CsA-IPT5* mRNA. The primers and the PCR amplification program can be seen in Table S3. The PCR program was: 94 °C for 5 min, followed by 35 cycles of 94 °C for 30 s, 59 °C for 30 s, and 72 °C for 30 s, and then a 10 min extension at 72 °C.

### Production of the CsA-IPT5-specific polyclonal antibody and western blot analysis

Young adult New Zealand white rabbits were selected. The first antigen concentration was 1 mg/ml, 0.5 ml/ rabbit, two-free - four free antigen concentration in half, the same dose. The first immunization was made with complete adjuvant. The injections two to four were made with incomplete adjuvant. The collected serum was tested using ELISA. The purified antibodies were detected using ELISA and western blot, respectively. An anti-CsA-IPT5 peptide antibody was purified with antigen affinity purification (Hua An Bioscience Technology, Hangzhou, China).

Total protein of the *N. benthamiana* leaves and tea plant tissues was extracted using a plant protein extraction kit containing a protease inhibitor cocktail. The GFP protein level in *N. benthamiana* leaves was measured by Western Blotting. The first antibody GFP (Abcam, ab183734) and the goat anti-Rabbit IgG(H + L)secondary antibody (Thermo Pierce, 31,210) were used here. The synthetic GFP constructs were listed in Figure S5.

The total protein of tea plant tissues was quantified using the BCA protein assay kit. For western-blot analysis, the extracted proteins were separated by 10% SDS-PAGE and transferred to PVDF membranes. The primary antibody (anti-CsA-IPT5 peptide antibody) and secondary antibody were hybridized successively. The signal was detected using the SuperSignal® West Dura Extended Duration Substrate.

### 6-BA treatment and the pruning treatment

For shrub-type mature tea garden in which only spring tea was picked up, tea plants usually need pruning twice a year. For the first pruning, tea trees were heavily pruned after spring tea in late April, and all the branch leaves that are approximately 50 cm above the ground are pruned; the second pruning was carried out around July 20.

The 6-BA treatment of tea plant was described in our previous report [38]. Specifically, mature tea plants of cv. LJ43 that were heavily pruned in late April were used. Working solutions of 200 mg L^− 1^ 6-benzyladine (6-BA) (J&K Scientific Ltd., Beijing, China) were prepared, and 0.1% (v/v) Tween 20 was added before spraying. When the apical buds and two to three leaves below the bud grew out, the foliar portion of tea bushes was sprayed with 6-BA solutions. Control tea bushes were simultaneously sprayed with distilled H_2_O containing the same ratio of Tween 20.

For the second pruning experiment, the mature tea plants cv. ZC108 which were heavily pruned in late April were used. The second pruning was conducted at a height that was 20 cm higher than the last cut surface on July 25, 2019. Control tea bushes were not pruned. Three tissues of the first node below the cuttings, including the internode, stem node and axillary bud, were sampled at 0 h, 12 h, 24 h, 36 h, 48 h, 4 d, 8 d, and 16 d after pruning.

For the studies of 6-BA and pruning treatments, the samples both were collected from more than 10 trees as one biological replicate, frozen in liquid N_2_ immediately after sampling, and stored at − 80 °C until analysis.

### Measurement of iP- and tZ-type CTK contents

The detection of iP- and *t*Z-type CTK contents was carried out using Agilent 1290 Infinity LC Ultra Performance Liquid Chromatography (UHPLC, Agilent) and 5500 QTRAP mass spectrograph (MS, AB SCIEX) (UHPLC-MS) (Shanghai Applied Protein Technology Co. Ltd., China).

### Statistical analysis

Data were analyzed using Statistica (SAS Institute Inc., http://www.statsoft.com). Data were analyzed by one-way ANOVA; if the ANOVA analysis was significant (*P <* 0.05), Duncan’s multiple range test was used to detect the significant differences between/among groups. Differences between the control and treatment were determined by Student’s *t*-tests.

## Supplementary Information

**Additional file 1: Figure S1.** The 3′ RACE and 5′ RACE PCR amplification of tea plant *CsA-IPT*. **Figure S2.** Analysis of the ORF and regulatory elements of the full-length cDNA of *CsA-IPT*. **Figure S3.** Multiple sequence alignment of the nucleotide sequences among the cloned full-length *CsA-IPT* cDNA in cv. Longjing 43 and the reported *CsA-IPT5* in cv. Shuchazao, and phylogenetic tree construction of A-IPT in several plant species. **Figure S4.** Alignment of 5′ RACE and 3′ RACE AS of tea plant *CsA-IPT5*, respectively. **Figure S5.** The nucleotide sequences and specific motifs in the GFP fluorescence of *CsA-IPT5* UTR splice variants. **Figure S6.** The qPCR detection of GFP. Data are means ±standard deviation (SD) (*n* = 6). In each figure, letters indicate significant differences among the GFP constructs (*P <* 0.05, Duncan’s multiple range test). **Figure S7.** Amplification of the different primer pairs for the 3′ UTR-AS and 5′ UTR-AS of *CsA-IPT5.*

**Additional file 2: Table S1.** The RACE PCR primers for *CsA-IPT* gene. **Table S2.** Primer combination used for RACE PCR of *CsA-IPT* gene. **Table S3.** Primer sequences used for real-time quantitative PCR (qPCR) of *CsA-IPT5* AS variants and housekeeping gene of tea plant. **Table S4.** The total CsA-IPT5 transcipts and the expression ratio of each splice variant in the internode induced by 6-BA application.

**Additional file 3.**

**Additional file 4.**

## Data Availability

All the data and materials that are required to reproduce these findings can be shared by contacting the first author, zhanglp2016@tricaas.com (ZLP).
