# Supplementary material for: A novel adenylate isopentenyltransferase 5 regulates shoot branching via the ATTTA motif in Camellia sinensis
Source: BMC Plant Biol. 2021 Nov 9;21:521. doi: 10.1186/s12870-021-03254-5 (PMC8577036; doi:10.1186/s12870-021-03254-5)
Supplement: Supplementary file 1 — Additional file 1: Figure S1. The 3′ RACE and 5′ RACE PCR amplification of tea plant CsA-IPT. Figure S2. Analysis of the ORF and regulatory elements of the full-length cDNA of CsA-IPT. Figure S3. Multiple sequence alignment of the nucleotide sequences among the cloned full-length CsA-IPT cDNA in cv. Longjing 43 and the reported CsA-IPT5 in cv. Shuchazao, and phylogenetic tree construction of A-IPT in several plant species. Figure S4. Alignment of 5′ RACE and 3′ RACE AS of tea plant CsA-IPT5, respectively. Figure S5. The nucleotide sequences and specific motifs in the GFP fluorescence of CsA-IPT5 UTR splice variants. Figure S6. The qPCR detection of GFP. Data are means ±standard deviation (SD) (n = 6). In each figure, letters indicate significant differences among the GFP constructs (P < 0.05, Duncan’s multiple range test). Figure S7. Amplification of the different primer pairs for the 3′ UTR-AS and 5′ UTR-AS of CsA-IPT5. [file 12870_2021_3254_MOESM1_ESM.docx]

**Figure S1.** The 3' RACE and 5' RACE PCR amplification of tea plant *CsA-IPT*. A: The 3' RACE PCR amplification of tea plant *CsA-IPT*. B: The 5' RACE PCR amplification of tea plant *CsA-IPT*. Lane A: the first PCR amplification; lane B: the second PCR amplification; lanes 1-6 correspond to 50.2, 53, 57, 59.9, 64.2, and 67.5 ℃, respectively. M: DL2000 plus and the lane represents 5000, 3000, 2000, 1000, 750, 500, 250, and 100 bp, respectively, from top to bottom.

1 ATACATACATACCAAGAAGGAAAAAACC**ATTTA**GTACTACCTTTGTTTGATACACAT**ATT**

61 **TA**CACACACTTTCCCAAAATTTCCAAACAAAATTCTTGGCCACCTTGTTGCATCCGTGAT

121 **ATTTA**AGCAGGTTTTTGC**ATTTA**TTATGAGAATTTCATTCTCACCTGCTTGCAAACAAGT

**M R I S F S P A C K Q V**

181 GGCGCAACCCCTTGTAAATTTTCCGGCCGGAGGAATTAATGATCCGTTCATCCTCCGGCA

**13 A Q P L V N F P A G G I N D P F I L R Q**

241 ACGGAAGAAAGAGAAGGTGGTTGTGGTGATTGGTGCAACTGGGACAGGAAAATCAAGACT

**33 R K K E K V V V V I G A T G T G K S R L**

301 TGCAATCGACTTGGCGACCCGTTTACCCGCGGAGATTATAAACTCGGACAAAATGCAGGT

**53 A I D L A T R L P A E I I N S D K M Q V**

361 CTACAAAGGGCTTGATATAGCCACCAACAAAGTAACCGACGAAGGGTGCCGCAGCGTGCC

**73 Y K G L D I A T N K V T D E G C R S V P**

421 ACATC**ATTTA**CTCGGGATTGTAGATCCAAACGCGGACTTCAACGCCACCGATTTTCGATA

**93 H H L L G I V D P N A D F N A T D F R Y**

481 CCTGGCATTGCACGCTTGCGATTCGATCGTACGGCGGAACGGGCTTCCAATCATCGCGGG

**113 L A L H A C D S I V R R N G L P I I A G**

541 TGGGTCCAATTCCTACATCAAGGCTTTGGTCAGCGATGATTTTGAATTCCGGTCGAAATA

**133 G S N S Y I K A L V S D D F E F R S K Y**

601 TGATTGTTGCTTTTTATGGGTTGATGTAAAATTACGCGTGCTTCACTCTTTTGTCTCGGA

**153 D C C F L W V D V K L R V L H S F V S E**

661 GCGGGTTGATCGGATGGTGGAAGCGGGACTGGTAGACGAGGTTCGAGCAATGTTTGACCC

**173 R V D R M V E A G L V D E V R A M F D P**

721 GAATGCTGATTATAAAGTTGGAATCAGACGAGCTATCGGAGTTCCAGAAATGGATCGGTT

**193 N A D Y K V G I R R A I G V P E M D R F**

781 TCTACGATCCGAATCCGACCCGAATGTCGATAGCCAGACCAGAGCTCGCCTACTTAAACA

**213 L R S E S D P N V D S Q T R A R L L K Q**

841 AGCCATTGAAGAAATCAAGTCCAACACTCGCGACTTGGCTTGTCGTCAGCGGAAGAACAT

**233 A I E E I K S N T R D L A C R Q R K N I**

901 TTTGAGCCTCCAAACCCAACTGGGTTTGGGTTTGGGTTTGGATTTGGGTTTGGGCCTGGG

**253 L S L Q T Q L G L G L G L D L G L G L G**

961 TCTGGGTCCAGAATGGAGCATGCTCCACCGCCTCGACGCCACGGAGGTGTTCCTCAGGCG

**273 L G P E W S M L H R L D A T E V F L R R**

1021 AGGCGCCGCCGCCGCAGCCGAGGCGTGGGAGAGGCTGGTGGCGGAGCCGAGCACCATCAT

**293 G A A A A A E A W E R L V A E P S T I I**

1081 CGTGGACCGGTTCCTCCACGAAAAAGACGACCAACAAGATTTCATGGCCGCCATCGCCAC

**313 V D R F L H E K D D Q Q D F M A A I A T**

1141 CCCGGCTTCCATCTCCGCCGCCGCTGTCGCCGCCACAACTCGGTAAAGCTGCTGGGGCTC

**333 P A S I S A A A V A A T T R ***

1201 ATTAAATGTACGGTAGAAATGACACGTGGGATGAGGACAAAATGATCATTCGATAGAGAC

1261 CTCACAAAACACCTCCTCTAAACTAATTTGAGCC**ATTTA**TGTTACTGGAGATATAATCTT

1321 TAAAAAAATTGCACACCAATAGTAAGCTTCCGTTTGGGAAGTGGGTTCGTTTTTTGACTT

1381 TTTTAATTTTTTTGACTATTTTAGTTTGGGCTAAAAAATTTG**ATTTA**GAAAGGATTTTTG

1441 GCTTTTTAATTTTTTTGACTTTTTTTTATGAGAGAAAAGTATAATGATTATGGGTTGGAG

1501 GTAATGTTTTGTTTGTTGCGGCAAAAAAAAAAAAAAAAAAAAAAAAA

**Figure S2.** Analysis of the ORF and regulatory elements of the full-length cDNA of *CsA-IPT*. The 5' UTR was highlighted with light grey. The blue letters represent ORF. The 3' UTR was highlighted with underline. The unstable ATTTA motifs were highlighted with single underline, bold letters, and dot underline.

**Figure S3.** Multiple sequence alignment of the nucleotide sequences among the cloned full-length *CsA-IPT* cDNAincv. Longjing 43 and the reported *CsA-IPT5* in cv. Shuchazao, and phylogenetic tree construction of A-IPT in several plant species. A: Multiple sequence alignment of the ORF sequences among the cloned *CsA-IPT* and the reported *CsA-IPT5* genes(TEA025674.1, XP_028094976.1, and XP_028094976.1); B: Multiple sequence alignment of the sequences in the 5' UTR and 3' UTR among the cloned *CsA-IPT* and the reported *CsA-IPT5* (XP_028094976.1 and XP_028094976.1), respectively; C: Phylogenetic analysis of A-IPT5 proteins from tea plant and other species. The phylogenetic tree was constructed on the basis of 15 amino acid sequences of *A-IPT5* genes through the UPGMA method by using MEGA software. The alignment of amino acid sequences was performed using the Neighbor-joining option of Clustal W.

**Figure S4.** Alignment of 5' RACE and 3' RACE AS of tea plant *CsA-IPT5*, respectively.Alignment of 3' RACE AS (A) and 5' RACE AS (B) of *CsA-IPT5*, respectively; C: Alignment of 3' RACE AS with the last two ATTTA motifs; D:Alignment of 5' RACE AS with the first four ATTTA motifs.

**(A-1) Gene: IPT 5AS1**

The sequence:
CgACgACAAgACCgTcaccGATCTTTCCCATATACATACATACCAAGAAGGAAAAAACCATTTAGTACTACCTTTGTTTGATTCACATATTTACACACACTTTCCCAAAATTTCCAAACAAAATTCTTGGCCACCTTGTTGCATCCGTGATATTTAAGGTTTTTGCATTTATTcgAcGGcTctTcTccTc

**(A-2) Gene: IPT 5AS1 M4-1&2**

The sequence:
CgACgACAAgACCgTcaccGATCTTTCCCATATACATACATACCAAGAAGGAAAAAACCGCCCGGTACTACCTTTGTTTGATTCACATGCCCGCACACACTTTCCCAAAATTTCCAAACAAAATTCTTGGCCACCTTGTTGCATCCGTGATATTTAAGGTTTTTGCATTTATTcgAcGGcTctTcTccTc

**(A-3) Gene: IPT 5AS1 M4-1&2&3**

The sequence:
CgACgACAAgACCgTcaccGATCTTTCCCATATACATACATACCAAGAAGGAAAAAACCGCCCGGTACTACCTTTGTTTGATTCACATGCCCGCACACACTTTCCCAAAATTTCCAAACAAAATTCTTGGCCACCTTGTTGCATCCGTGATGCCCGAGGTTTTTGCATTTATTcgAcGGcTctTcTccTc

**(B-1) Gene: IPT 5AS2**

The sequence:
CgACgACAAgACCgTcaccATACATACATACCAAGAAGGAAAAAACCATTTAGTACTACCTTTGTTTGATACACATATTTACACACACTTTCCCAAAATTTCCAAACAAAATTCTTGGCCACCTTGTTGCATCCGTGATATTTAAGCAGGTTTTTGCATTTATTcgAcGGcTctTcTccTc

**(B-2) Gene: IPT 5AS2 M4-1&2**

The sequence:
CgACgACAAgACCgTcaccATACATACATACCAAGAAGGAAAAAACCGCCCGGTACTACCTTTGTTTGATACACATGCCCGCACACACTTTCCCAAAATTTCCAAACAAAATTCTTGGCCACCTTGTTGCATCCGTGATATTTAAGCAGGTTTTTGCATTTATTcgAcGGcTctTcTccTc

**(B-3) Gene: IPT 5AS2 M4-1&2&3**

The sequence:
CgACgACAAgACCgTcaccATACATACATACCAAGAAGGAAAAAACCGCCCGGTACTACCTTTGTTTGATACACATGCCCGCACACACTTTCCCAAAATTTCCAAACAAAATTCTTGGCCACCTTGTTGCATCCGTGATGCCCGAGCAGGTTTTTGCATTTATTcgAcGGcTctTcTccTc

**(C-1) Gene: IPT 5AS3**

The sequence:
CgACgACAAgACCgTcaccCCTCATTAATACTATCATTTCTTTGTACATCTCCCTGGAGTTTCTATGAAAAGTTCACTTTGTGTTCTTATATTTGCTTTTTCACAATCTGTCAGCTGGTTTTTGCATTTATTcgAcGGcTctTcTccTc

**(C-2) Gene: IPT 5AS3 M1**

The sequence:
CgACgACAAgACCgTcaccCCTCATTAATACTATCATTTCTTTGTACATCTCCCTGGAGTTTCTATGAAAAGTTCACTTTGTGTTCTTATATTTGCTTTTTCACAATCTGTCAGCTGGTTTTTGCGCCCGTTcgAcGGcTctTcTccTc

**(D-1) Gene: IPT 3AS1**

The sequence: CsA-IPT5 3′ UTR-AS1

CgACgACAAgACCgTcaccAGCTGCTGGGGCTC**ATTAAA**TGTACGGTAGAAATGACACGTGGGATGAGGACAAAATGATCATTCGATAGAGACCTCACAAAACACCTCCTCTAAACTAATTTGAGCC**ATTTA**TGTTACTGGAGATATAATCTTTAAAAAAATTGCACACCAATAGTAAGCTTCCGTTTGGGAAGTGGGTTCGTTTTTTGACTTTTTTAATTTTTTTGACTATTTTAGTTTGGGCTAAAAAATTTG**ATTTA**GAAAGGATTTTTGGCTTTTTAATTTTTTTGACTTTTTTTTATGAGAGAAAAGTATAATGATTATGGGTTGGAGGTAATGTTTTGTTTGTTGCGGCAAAAAAAAAAAAAAAAAAAAAAAAcgAcGGcTctTcTccTc

**(D-2) Gene: IPT 3AS1 M2-1**

The sequence:

CgACgACAAgACCgTcaccAGCTGCTGGGGCTC**ATTAAA**TGTACGGTAGAAATGACACGTGGGATGAGGACAAAATGATCATTCGATAGAGACCTCACAAAACACCTCCTCTAAACTAATTTGAGCCGCCCGTGTTACTGGAGATATAATCTTTAAAAAAATTGCACACCAATAGTAAGCTTCCGTTTGGGAAGTGGGTTCGTTTTTTGACTTTTTTAATTTTTTTGACTATTTTAGTTTGGGCTAAAAAATTTG**ATTTA**GAAAGGATTTTTGGCTTTTTAATTTTTTTGACTTTTTTTTATGAGAGAAAAGTATAATGATTATGGGTTGGAGGTAATGTTTTGTTTGTTGCGGCAAAAAAAAAAAAAAAAAAAAAAAAcgAcGGcTctTcTccTc

**(D-3) Gene: IPT 3AS1 M2-1&2**

The sequence:

CgACgACAAgACCgTcaccAGCTGCTGGGGCTC**ATTAAA**TGTACGGTAGAAATGACACGTGGGATGAGGACAAAATGATCATTCGATAGAGACCTCACAAAACACCTCCTCTAAACTAATTTGAGCCGCCCGTGTTACTGGAGATATAATCTTTAAAAAAATTGCACACCAATAGTAAGCTTCCGTTTGGGAAGTGGGTTCGTTTTTTGACTTTTTTAATTTTTTTGACTATTTTAGTTTGGGCTAAAAAATTTGGCCCGGAAAGGATTTTTGGCTTTTTAATTTTTTTGACTTTTTTTTATGAGAGAAAAGTATAATGATTATGGGTTGGAGGTAATGTTTTGTTTGTTGCGGCAAAAAAAAAAAAAAAAAAAAAAAAcgAcGGcTctTcTccTc

**(E-1) Gene: IPT 3AS2**

The sequence:

CgACgACAAgACCgTcaccAGCTGCTGGGGCTC**ATTAAA**TGTACGGTAGAAATGACACGTGGGATGAGGACAAAATGATCATTCGATAGAGACCTCACAAAACACCTCCTCTAAACTAATTTGAGCC**ATTTA**TGTTACTGGAGATATAATCTTTAAAAAAATTGCACACCAATAGTAAGCTTCCGTTTGGGAAGTGGGTTCGTTTTTTGACTTTTTTAATTTTTTTGACTATTTTAGTTTGGGCTAAAAAATTTG**ATTTA**GAAAGGATTTTTGGCTTTTCGAAAAAAAAAAA**AATAAA**AAAAAAAAAAAAAAcgAcGGcTctTcTccTc

**(E-2) Gene: IPT 3AS2 M2-1**

The sequence:

CgACgACAAgACCgTcaccAGCTGCTGGGGCTC**ATTAAA**TGTACGGTAGAAATGACACGTGGGATGAGGACAAAATGATCATTCGATAGAGACCTCACAAAACACCTCCTCTAAACTAATTTGAGCCGCCCGTGTTACTGGAGATATAATCTTTAAAAAAATTGCACACCAATAGTAAGCTTCCGTTTGGGAAGTGGGTTCGTTTTTTGACTTTTTTAATTTTTTTGACTATTTTAGTTTGGGCTAAAAAATTTG**ATTTA**GAAAGGATTTTTGGCTTTTCGAAAAAAAAAAA**AATAAA**AAAAAAAAAAAAAAcgAcGGcTctTcTccTc

**(E-3) Gene: IPT 3AS2 M2-1&2**

The sequence:

CgACgACAAgACCgTcaccAGCTGCTGGGGCTC**ATTAAA**TGTACGGTAGAAATGACACGTGGGATGAGGACAAAATGATCATTCGATAGAGACCTCACAAAACACCTCCTCTAAACTAATTTGAGCCGCCCGTGTTACTGGAGATATAATCTTTAAAAAAATTGCACACCAATAGTAAGCTTCCGTTTGGGAAGTGGGTTCGTTTTTTGACTTTTTTAATTTTTTTGACTATTTTAGTTTGGGCTAAAAAATTTGGCCCGGAAAGGATTTTTGGCTTTTCGAAAAAAAAAAA**AATAAA**AAAAAAAAAAAAAAcgAcGGcTctTcTccTc

**Figure S5.** The nucleotide sequences and specific motifs in the GFP fluorescenceof *CsA-IPT5* UTR splice variants.


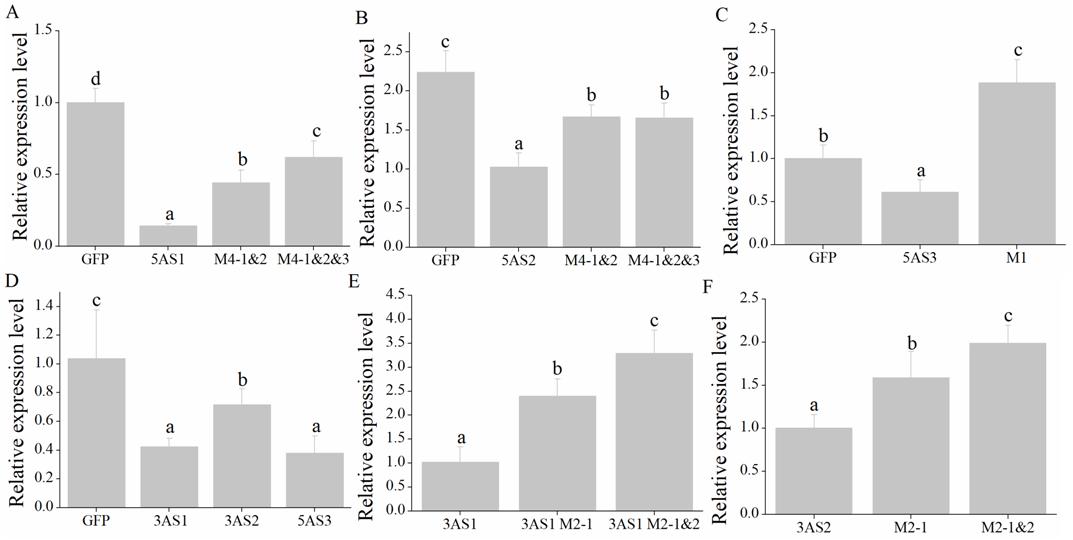


**Figure S6.** The qPCR detection of GFP. Data are means ±standard deviation (SD) (n=6). In each figure, letters indicate significant differences among the GFP constructs (*P <*0.05, Duncan’s multiple range test).

**Figure S7.** Amplification of the different primer pairs for the 3′ UTR AS and 5′ UTR AS of *CsA-IPT5*.A: Amplification of the different primers for the 3′ UTR AS of *CsA-IPT5*. Lane 4, 5, 6 and 7 correspond to the primer pairs 5AS4F+5ASR (201bp), 3ASF+3AS4R (199 bp), 3ASF+ 3AS2R (304 bp) and 3ASF+3AS1R (427 bp), respectively; Lane 10 and 11 correspond to the primer pairs CsGAPDH F+ CsGAPDH R1 (166bp) and CsGAPDH F+CsGAPDH R2 (205bp), respectively; B: Amplification of the different primer pairs for the 5′ UTR AS of *CsA-IPT5*. Lane A correspond to the primer pairs 5AS1F+ 5ASR, which was used for detecting the 5AS1(247bp), and lane A1-A6 correspond to the amplification results at 50, 52, 54, 55, 57 and 63℃, respectively; lane B correspond to the primer pairs 5AS2F+5ASR, which was used for detecting the 5AS2 (247bp), and lane B1-B6 correspond to the amplification results at 50, 52, 54, 55, 57 and 63℃, respectively; lane C correspond to the primer pairs 5AS3F+5ASR, which was used for detecting the 5AS3 (249 bp), and lane C1-C6 correspond to the amplification results at 50, 52, 54, 55, 57 and 63℃, respectively. M: DL1000 plus and the lane represents 1000, 750, 500, 250, and 100 bp, respectively, from top to bottom.
