# Supplementary material for: A novel adenylate isopentenyltransferase 5 regulates shoot branching via the ATTTA motif in Camellia sinensis
Source: BMC Plant Biol. 2021 Nov 9;21:521. doi: 10.1186/s12870-021-03254-5 (PMC8577036; doi:10.1186/s12870-021-03254-5)
Supplement: Supplementary file 2 — Additional file 2: Table S1. The RACE PCR primers for CsA-IPT gene. Table S2. Primer combination used for RACE PCR of CsA-IPT gene. Table S3. Primer sequences used for real-time quantitative PCR (qPCR) of CsA-IPT5 AS variants and housekeeping gene of tea plant. Table S4. The total CsA-IPT5 transcipts and the expression ratio of each splice variant in the internode induced by 6-BA application. [file 12870_2021_3254_MOESM2_ESM.doc]

**Table S1.** The RACE PCR primers for *CsA-IPT* gene

| Primer | 5'→3' | Design purpose |
| --- | --- | --- |
| CsIPT-F query_L1 | GATCCAAACGCGGACTTCAA | The forward primer for qPCR of *CsA-IPT* which was used for the first 3' RACE-PCR |
| CsIPT-3R2 | CCAATCATCGCGGGTGGGT | It was used for the second 3' RACE PCR |
| CsIPT-5R1 | GGTGTTTTGTGAGGTCTCTATC | It was used for the first 5' RACE-PCR |
| CsIPT-5R2 | GGAATTGGACCCACCCGCGA | It was used for the second 5' RACE-PCR |

**Table S2.** Primer combination used for RACE PCR of *CsA-IPT* gene

| Primer combination | | PCR amplification | Expected length (bp) | Annealing temperature (℃) |
| --- | --- | --- | --- | --- |
| CsIPT-F query_L1+3' adaptor primer (3AP) | | The first  3' RACE-PCR | Unknown, ~1 kb | 59 |
| CsIPT-3R2+3' adaptor primer (3AP) | The second  3' RACE-PCR | | Unknown, it is ~1 kb and is 84 bp shorter than the product of ‘CsIPT-F query_L1+ 3AP’ | 59 |
| CsIPT-5R1+5' adaptor primer (5AP) | The first  5' RACE-PCR | | Unknown, ~1.2 kb | 59 |
| CsIPT-5R2+5' adaptor primer (5AP) | The second  5' RACE-PCR | | Unknown, it is 0.7 kb shorter than the product  of ‘CsIPT-5R1+5A’ | 59 |

**Table** **S3. Primer sequences used for real-time quantitative PCR (qPCR) of *CsA-IPT5* AS variants and housekeeping gene of tea plant**

| **Primer** | **5'→3'** | | **Purpose of design** | **PCR product (bp)** | **The corresponding lane** |
| --- | --- | --- | --- | --- | --- |
| A-IPT 5AS1F | GTTGCATCCGTGATATTTAAGGT | The forward primer for detecting 5′ UTR AS1 | | A-IPT 5AS1F+ A-IPT 5ASR=247 | Fig. S12, lane 1; Fig.2, lane A1~A6 |
| A-IPT 5AS2F | GCATCCGTGATATTTAAGCAGGT | The forward primer for detecting 5′ UTR AS2 | | A-IPT 5AS2F+ A-IPT 5ASR=247 | Fig. S12, lane 2; Fig.2, lane B1~B6 |
| A-IPT 5AS3F | GCTTTTTCACAATCTGTCAGCTG | The forward primer for detecting 5′ UTR AS3 | | A-IPT 5AS3F+ A-IPT 5ASR=249 | Fig. S12, lane 3; Fig. 2, lane C1~C6 |
| A-IPT 5AS4F | CATTCTCACCTGCTTGCAAACA | The forward primer for detecting the total expression of *CsA-IPT5* | | A-IPT 5AS4F+ A-IPT 5ASR=201 | Fig. S6A, lane 4. It was used for detecting the total expression |
| A-IPT 5ASR | GCATTTTGTCCGAGTTTATAATCTC | The reverse primer for detecting different AS in 5′ UTR and the total expression of *CsA-IPT5*, and the different forward primers all combine with it, respectively. | |  |  |
| A-IPT 3ASF | CATCATCGTGGACCGGTT | The forward primer for detecting the expression of different 3′ UTR AS, and the different reverse primer of 3′ UTR AS all combine with it, respectively. | |  |  |
| A-IPT 3AS1R | CCTCCAACCCATAATCATTATACTT | The reverse primer for detecting 3′ UTR AS1 | | A-IPT 3ASF+ A-IPT 3AS1R=427 | Fig. S12, lane 7 |
| A-IPT 3AS2R | GTCAAAAAACGAACCCACTTCC | The reverse primer for detecting 3′ UTR AS2 | | A-IPT 3ASF+ A-IPT 3AS2R=304 | Fig. S12, lane 6 |
| A-IPT 3AS4R | GGTGTTTTGTGAGGTCTCTATC | The reverse primer for detecting the total expression of *A-IPT5* | | A-IPT 3ASF+ A-IPT 3AS4R=199 | Fig. S12, lane 5 |
| A-IPT 3ASF2 | CACAACTCGGTAAAGCTGCT | The forward primer 2 for detecting 3′ UTR AS1 and 3′ UTR AS2 | | A-IPT 3ASF2+ A-IPT 3AS1R=328 | Fig. S12, lane 8 |
|  |  |  |  | A-IPT 3ASF2+ A-IPT 3AS2R=205 | Fig. S12, lane 9 |
| CsGAPDH F | GAGACTGGAGCCGAATTCATT | The forward primer for detecting housekeeping gene GAPDH | |  |  |
| CsGAPDH R1 | GATCTGGCTTGTAATCCTTCTCA | The reverse primer 1 for detecting GAPDH gene | | CsGAPDH F+ CsGAPDH R1=166 | Fig. S12, lane 10 |
| CsGAPDH R2 | GACAGTTGGTAGTGCAGCTA | The reverse primer 2 for detecting GAPDH gene | | CsGAPDH F+ CsGAPDH R2=205 | Fig. S12, lane 11 |

**Table S4.** The total CsA-IPT5 transcipts and the expression ratio of each splice variant in the internode induced by 6-BA application.

|  | | 0h | 12h | 24h | 3d | 5d | 9d |
| --- | --- | --- | --- | --- | --- | --- | --- |
| Total transcipts | Control | 0.0036±0.00028ab | 0.0031±0.00068a | 0.0032±0.00053a | 0.0040±0.0010ab | 0.0045±0.00060b | 0.0031±0.00035a |
|  | 6-BA | 0.0036±0.00028b | 0.0017±0.00046a | 0.0044±0.00017b | 0.0059±0.0016c | 0.0077±0.0010d | 0.0021±0.00028a |
| 5AS1 | Control | 0.28±0.016a | 0.71±0.037b | 0.89±0.094c | 0.89±0.093c | 0.74±0.061bc | 0.84±0.14bc |
|  | 6-BA | 0.28±0.016a | 1.14±0.108d | 1.12±0.098d | 0.86±0.018c | 0.68±0.085b | 0.75±0.056bc |
| 5AS2 | Control | 0.24±0.027a | 1.98±0.158c | 1.84±0.157c | 1.21±0.17b | 1.37±0.024b | 1.23±0.18b |
|  | 6-BA | 0.24±0.027a | 2.51±0.246c | 2.57±0.41c | 1.17±0.032b | 1.30±0.12b | 1.32±0.25b |
| 5AS3 | Control | 0.41±0.028a | 0.46±0.021ac | 0.58±0.052b | 0.56±0.095b | 0.54±0.018bc | 0.43±0.043a |
|  | 6-BA | 0.41±0.028bc | 0.45±0.079c | 0.16±0.027a | 0.33±0.070b | 0.45±0.061c | 0.44±0.049c |
| 3AS1 | Control | 2.06±0.11a | 2.76±0.27a | 3.98±0.68b | 5.84±0.59c | 6.89±0.201d | 7.47±0.26d |
|  | 6-BA | 2.06±0.11a | 5.18±0.58bc | 5.92±0.33c | 5.99±0.69c | 5.82±0.98c | 4.57±0.86b |
| 3AS2 | Control | 42.7±7.01a | 42.0±4.35a | 43.8±3.01a | 41.2±2.92a | 74.7±3.05a | 49.3±3.32a |
|  | 6-BA | 42.7±7.01ab | 44.90±4.54ab | 42.4±2.57ab | 40.6±1.69ab | 37.3±6.36a | 47.4±4.75b |

As for the total *CsA-IPT5* transcipts or the expression ratio of each *CsA-IPT5* splice variant, the differences of the gene expression among different time points were analyzed, respectively. Data shown were means ± SD (n = 6). Letters indicate significant differences of each index among different time points (*P <*0.05, Duncan’s multiple range test).
