# Supplementary material for: A novel adenylate isopentenyltransferase 5 regulates shoot branching via the ATTTA motif in Camellia sinensis
Source: BMC Plant Biol. 2021 Nov 9;21:521. doi: 10.1186/s12870-021-03254-5 (PMC8577036; doi:10.1186/s12870-021-03254-5)
Supplement: Supplementary file 3 — Additional file 3. [file 12870_2021_3254_MOESM3_ESM.docx]

# Primer pairs: 5AS1F+5ASR (5AS1), 5AS2F+5ASR (5AS2), 5AS3F+5ASR (5AS3), 5AS4F+5ASR (5AS4), 3ASF+3AS1R (3AS1), 3ASF+3AS2R (3AS2), 3ASF+3AS4R (the total transcript), 3ASF+A-3AS4R (3AS4), CsGAPDH F+CsGAPDH R1 (GAPDH).

# The electrophoresis validation of PCR products are shown in the 3 pictures bllow:


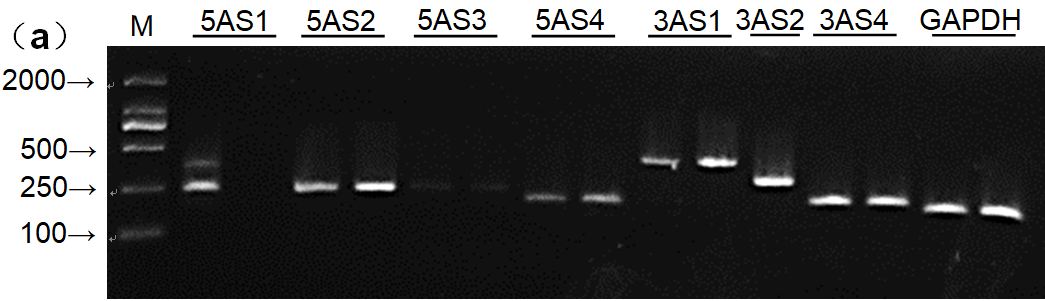


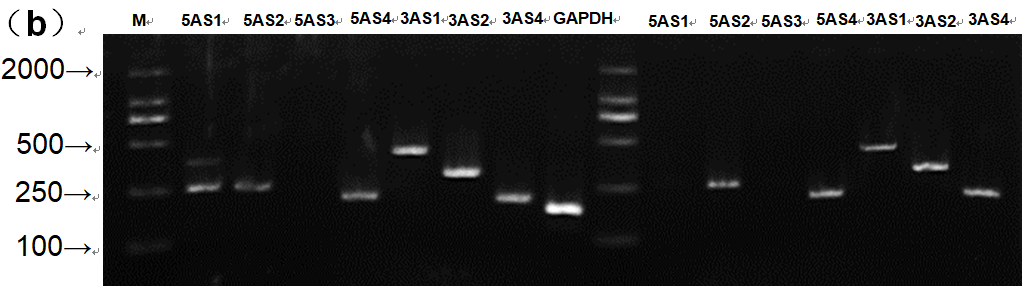


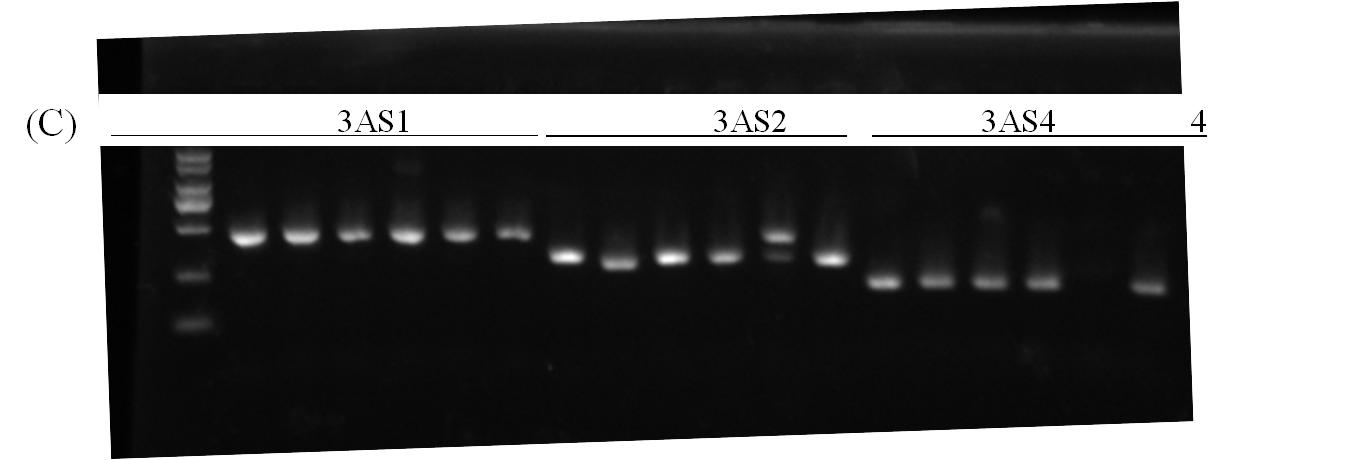


The above results showed that there are all amplified bands for each primer pair, and only the amplified band of 5AS3 is relatively weak. However, the amplified band of 5AS3 can still be recovered.

The sequences of monoclones cloned by the qPCR primers and the sequences between the front and back qPCR primers were sequenced and alignmented, and the results are as follows:

**1. Highly consistent sequences of 5AS1：**

>5AS1-e2

CTTGTTGCATCCGTGATATTTAAGGTTTTTGCATTTATTATGAGAATTTCATTCTCACCTGCTTGCAAACAAGTGGCACAACCCCTTGTAAATTTTCCGGCCGGAGGAATTAATGATCCGTTCATCCTCCGGCAACGGAAGAAAGAGAAGGTGGTTGTAGTGATTGGTGCAACTGGGACAGGAAAATCAAGACTTGCAATCGACTTGGCGACCCGTTTACCCGCGGAGATTATAAACTCGGACAAAATGCAAG

>5AS1-RACE2

CTTGTTGCATCCGTGATATTTAAGGTTTTTGCATTTATTATGAGAATTTTATTCTCACCTGCTTGCAAACAAGTGGCACAACCCCTTGTAAATTTTCCGGCCGGAGGAATTAATGATCCGTTCATCCTCCGGCAGCGGAAGAAAGAGAAGGTGGTTGTAGTGATTGGTGCAACTGGGACAGGAAAATCAAGACTTGCAATCGACTTGGCGACCCGTTTACCCGCGGAGATTATAAACTCGGACAAAATGCAAG


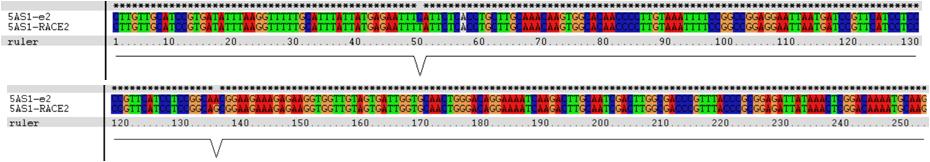


Note: ‘Double underline’ represent primer ‘5ASF+5AS1R’.

**2. Highly consistent sequences of 5AS2：**

>5AS2-b2

TTGCATCCGTGATATTTAAGCAGGTTTTTGCATTTATTATGAGAATTTCATTCTCACCTGCTTGCAAACAAGTGGCACAACCCCTTGTAAATTTTCCGGCCGGAGGAATTAATGATCCGTTCATCCTCCGGCAACGGAAGAAAGAGAAGGTGGTTGTAGTGATTGGTGCAACTGGGACAGGAAAATCAAGACTTGCAATCGACTTGGCGACCCGTTTACCCGCGGAGATTATAAACTCGGACAAAATGCA

>5AS2-RACE2

TTGCATCCGTGATATTTAAGCAGGTTTTTGCATTTATTATGAGAATTTCATTCTCACCTGCTTGCAAACAAGTGGCGCAACCCCTTGTAAATTTTCCGGCCGGAGGAATTAATGATCCGTTCATCCTCCGGCAACGGAAGAAAGAGAAGGTGGTTGTGGTGATTGGTGCAACTGGGACAGGAAAATCAAGACTTGCAATCGACTTGGCGACCCGTTTACCCGCGGAGATTATAAACTCGGACAAAATGCA


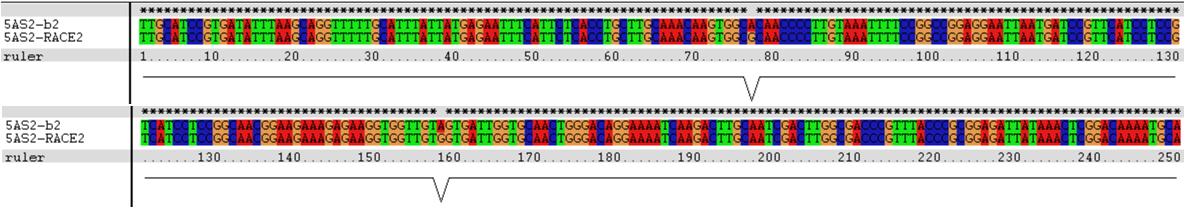


Note: ‘Double underline’ represent primer ‘5ASF+5AS2R’.

**3. Highly consistent sequences of 5AS3：**

>5AS3-a2

TTGCTTTTTCCAATCTGTCAGCAGGTTTTTGCATTTATTATGAGAATTTCATTCTCACCTGCTTGCAAACAAGTGGCACAACCCCTTGTAAATTTTCCGGCCGGAGGAATTAATGATCCGTTCATCCTCCGGCAACGGAAGAAAGAGAAGGTGGTTGTAGTGATTGGTGCAACTGGGACAGGAAAATCAAGACTTGCAATCGACTTGGCGACCCGTTTACCCGCGGAGATTATAAACTCGGACAAAATGCAAG

>5AS3-RACE2

TTGCTTTTTCACAATCTGTCAGCTGGTTTTTGCATTTATTATGAGAATTTCATTCTCACCTGCTTGCAAACAAGTGGCACAACCCCTTGTAAATTTTCCGGCCGGAGGAATTAATGATCCGTTCATCCTCCGGCAACGGAAGAAAGAGAAGGTGGTTGTAGTGATTGGTGCAACTGGGACAGGAAAATCAAGACTTGCAATCGACTTGGCGACCCGTTTACCCGCGGAGATTATAAACTCGGACAAAATGCAAG


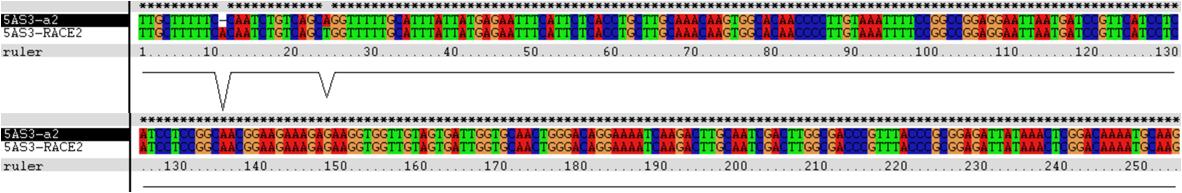


Note: ‘Double underline’ represent primer ‘5ASF+5AS3R’.

**4. Alignment of the senquences between primer 3ASF+3AS1R and cloned 3AS1-4**

>the senquences between primer 3ASF+3AS1R

CATCATCGTGGACCGGTTCCTCCACGAAAAAGACGACCAACAAGATTTCATGGCCGCCATCGCCACCCCGGCTTCCATCTCCGCCGCCGCTGTCGCCGCCACAACTCGGTAAAGCTGCTGGGGCTCATTAAATGTACGGTAGAAATGACACGTGGGATGAGGACAAAATGATCATTCGATAGAGACCTCACAAAACACCTCCTCTAAACTAATTTGAGCCATTTATGTTACTGGAGATATAATCTTTAAAAAAATTGCACACCAATAGTAAGCTTCCGTTTGGGAAGTGGGTTCGTTTTTTGACTTTTTTAATTTTTTTGACTATTTTAGTTTGGGCTAAAAAATTTGATTTAGAAAGGATTTTTGGCTTTTTAATTTTTTTGACTTTTTTTTATGAGAGAAAAGTATAATGATTATGGGTTGGAGG

> 3AS1-4, the senquences of cloned 3AS1 using primer 3ASF+3AS1R

AAATCATAGGGCGATTGATTTAGCGGCCGCGAATTCGCCCTTCATCATCGTGGACCGGTTCCTCCACGAAAAAGACGACCAACAAGATTTCATGGCCGCCATCGCCACCCCGGCTTCCATCTCCGCCGCCGCTGTCGCCGCCACAACTCGGTAAAGCTGCTGGGGCTCATTAAATGTACGGTAGAAATGACACGTGGGATGAGGACAAAATGATCATTCGATAGAGACCTCACAAAACACCTCCTCTAAACTAATTTGAGCCATTTATGTTACTGGAGATATAATCTTTAAAAAAATTGCACACCAATAGTAAGCTTCCGTTTGGGAAGTGGGTTCGTTTTTTGACTTTTTTAATTTTTTTGACTATTTTAGTTTGGGCTAAAAAATTTGATTTAGAAAGGATTTTTGGCTTTTTAATTTTTTTGACTTTTTTTTATGAGAGAAAAGTATAATGATTATGGGTTGGAGGAAGGGCGAATTCGTTTAAACCTGCAGGACTAGTACCTTTAGTGAGGGTTAATTCTGAGCTTGGCGTAATCATGGTCATAGCTGTTTCCTGTGTGAAATTGTTATCCGCTCACAATTCCACACAACATACGAGCCGGAAGCATAAAGTGTAAAGCCTGGGGTGCCTAATGAGTGAGCTAACTCACATTAATTGCGTTGCGCTCACTGCCCGCTTTCCAGTCGGGAAACCTGTCGTGCCAGCTGCATTAATGAATCGGCCAACGCGCGGGGAGAGGCGGTTTGCGTATTGGGCGCTCTTCCGCTTCCTCGCTCACTGACTCGCTGCGCTCGGTCGTTCGGCTGCGGCGAGCGGTATCAGCTCACTCAAAGGCGGTAATACGGTTATCCACAGAATCAGGGGATAACGCAGGAAAGAACATGTGAGCAAAAGGCCAGCAAAAGGCCAGGAACCGTAAAAAGGCCGCGTTGCTGGCGTTTTTCCATAGGCTCCGCCCCCCTGACGAGCATCACAAAAATCGACGCTCAAGTCAGAGGTGGCGAAACCCGACAGGACTATAAAGATACCAGGCGTTTCCCCCTGGAAGCTCCCTCGTGCGCTCTCCTGTTCCGACCCTGCCGCTTACCGGATACCTGGCCGCCTTTCTCCC


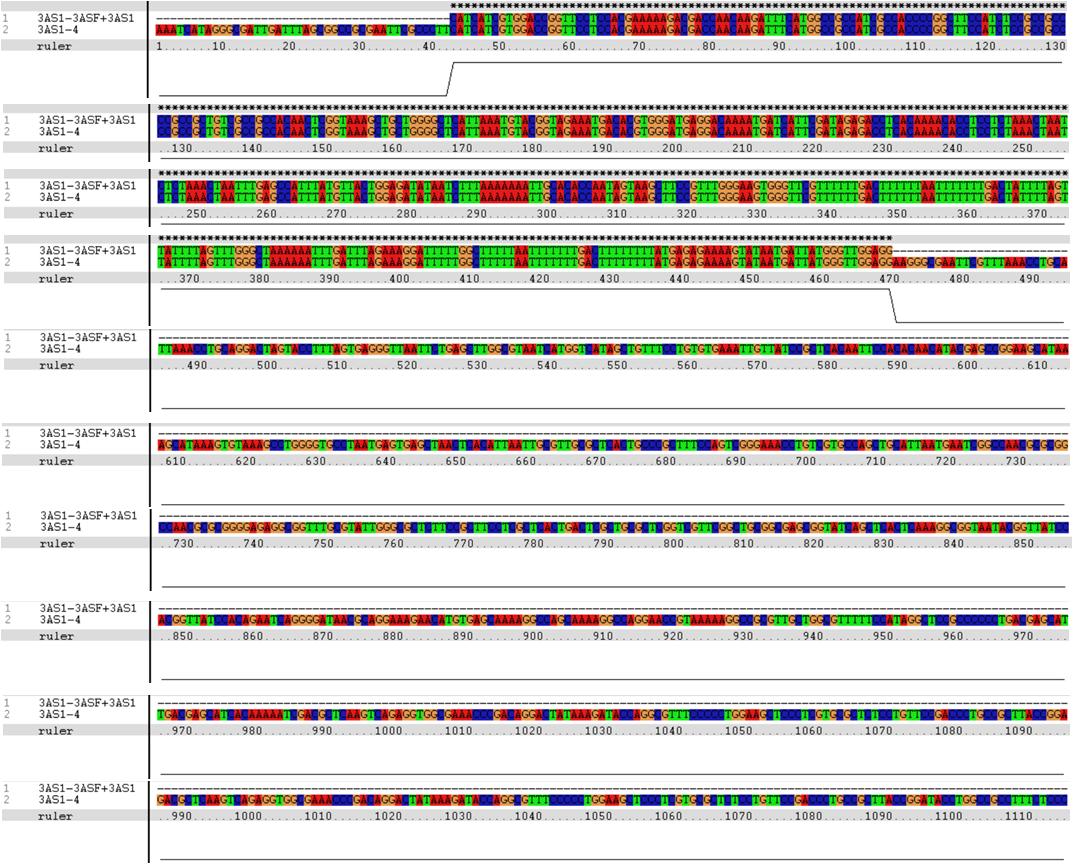


Figure 4 **Alignment of the senquences between primer 3ASF+3AS1 and cloned 3AS1-4：**

Note: ‘Double underline’ represent primer ‘3ASF+3AS1R’. ‘Yellow highlights’ represent aligned sequences.

**5. Alignment of the senquences between primer 3ASF+3AS2R and cloned 3AS2-2：**

>the senquences between primer 3ASF+3AS2R (3AS2-3ASF+3AS2R)

CATCATCGTGGACCGGTTCCTCCACGAAAAAGACGACCAACAAGATTTCATGGCCGCCATCGCCACCCCGGCTTCCATCTCCGCCGCCGCTGTCGCCGCCACAACTCGGTAAAGCTGCTGGGGCTCATTAAATGTACGGTAGAAATGACACGTGGGATGAGGACAAAATGATCATTCGATAGAGACCTCACAAAACACCTCCTCTAAACTAATTTGAGCCATTTATGTTACTGGAGATATAATCTTTAAAAAAATTGCACACCAATAGTAAGCTTCCGTTTGGGAAGTGGGTTCGTTTTTTGAC

>3AS2-2

GTAATCTATTAGGGCGATTGATTTAGCGGCCGCGAATTCGCCCTTCATCATCGTGGACCGGTTGACCCAACCCAAAATCCAACTACGAGCTTTTTAACTGCAACAACTTAAATATACGCTATTGGAGCTGGAATTACCGCGGCTGCTGGCACCAGACTTGCCCTCCAATGGATCCTCGTTAAGGGATTTAGATTGTACTCATTCCAATTACCAGACTCAAAGAGCCCGGTATTGTTATTTATTGTCACTACCTCCCCGTGTCAGGATTGGGTAATTTGCGCGCCTGCTGCCTTCCTTGGAAGTGGGTTCGTTTTTTGACAAGGGCGAATTCGTTTAAACCTGCAGGACTAGTACCTTTAGTGAGGGTTAATTCTGAGCTTGGCGTAATCATGGTCATAGCTGTTTCCTGTGTGAAATTGTTATCCGCTCACAATTCCACACAACATACGAGCCGGAAGCATAAAGTGTAAAGCCTGGGGTGCCTAATGAGTGAGCTAACTCACATTAATTGCGTTGCGCTCACTGCCCGCTTTCCAGTCGGGAAACCTGTCGTGCCAGCTGCATTAATGAATCGGCCAACGCGCGGGGAGAGGCGGTTTGCGTATTGGGCGCTCTTCCGCTTCCTCGCTCACTGACTCGCTGCGCTCGGTCGTTCGGCTGCGGCGAGCGGTATCAGCTCACTCAAAGGCGGTAATACGGTTATCCACAGAATCAGGGGATAACGCAGGAAAGAACATGTGAGCAAAAGGCCAGCAAAAGGCCAGGAACCGTAAAAAGGCCGCGTTGCTGGCGTTTTTCCATAGGCTCCGCCCCCCTGACGAGCATCACAAAAATCGACGCTCAAGTCAGAGGTGGCGAAACCCGACAGGACTATAAAGATACCAGGCGTTTCCCCCTGGAAGCTCCCTCGTGCGCTCTCCTGTTCCGACCCTGCCGCTTACCGGATACCT

> 3AS2-2, the senquences of cloned 3AS1 using primer 3ASF+3AS2

Note: ‘Double underline’ represent primer ‘3ASF+3AS2R’. ‘Yellow highlights’ represent aligned sequences.

**6. Alignment of the senquences between primer 3ASF+3AS4R and cloned 3AS4：**

>the senquences between primer 3ASF+3AS4R (**3AS4-**3ASF+3AS4R)

CATCATCGTGGACCGGTTCCTCCACGAAAAAGACGACCAACAAGATTTCATGGCCGCCATCGCCACCCCGGCTTCCATCTCCGCCGCCGCTGTCGCCGCCACAACTCGGTAAAGCTGCTGGGGCTCATTAAATGTACGGTAGAAATGACACGTGGGATGAGGACAAAATGATCATTCGATAGAGACCTCACAAAACACC

>3AS4-1

ATACATCTTAGGGGATTGATTTAGCGGCCGCGAATTCGCCCTTCATCATCGTGGACCGGTTCCTCCACGAAAAAGACGACCAACAAGATTTCATGGCCGCCATCGCCACCCCGGCTTCCATCTCCGCCGCCGCTGTCGCCGCCACAACTCGGTAAAGCTGCTGGGGCTCATTAAATGTACGGTAGAAATGACACGTGGGATGAGGACAAAATGATCATTCGATAGAGACCTCACAAAACACCAAGGGCGAATTCGTTTAAACCTGCAGGACTAGTACCTTTAGTGAGGGTTAATTCTGAGCTTGGCGTAATCATGGTCATAGCTGTTTCCTGTGTGAAATTGTTATCCGCTCACAATTCCACACAACATACGAGCCGGAAGCATAAAGTGTAAAGCCTGGGGTGCCTAATGAGTGAGCTAACTCACATTAATTGCGTTGCGCTCACTGCCCGCTTTCCAGTCGGGAAACCTGTCGTGCCAGCTGCATTAATGAATCGGCCAACGCGCGGGGAGAGGCGGTTTGCGTATTGGGCGCTCTTCCGCTTCCTCGCTCACTGACTCGCTGCGCTCGGTCGTTCGGCTGCGGCGAGCGGTATCAGCTCACTCAAAGGCGGTAATACGGTTATCCACAGAATCAGGGGATAACGCAGGAAAGAACATGTGAGCAAAAGGCCAGCAAAAGGCCAGGAACCGTAAAAAGGCCGCGTTGCTGGCGTTTTTCCATAGGCTCCGCCCCCCTGACGAGCATCACAAAAATCGACGCTCAAGTCAGAGGTGGCGAAACCCGACAGGACTATAAAGATACCAGGCGTTTCCCCCTGGAAGCTCCCTCGTGCGCTCTCCTGTTCCGACCCTGCCGCTTACCGGATACCTGTCCGCCTTTCTCCCTTCGGGAAGCGTGGCGCTTTCTCATAGCTCACGCTGTAGGTATCTCAGTTCGGTGTAGGTCGTTCGCTCCAAGCTGGGCTGTGTGCACGAACCCCCCGTTCAGCCCGACCGCTGCGCCTTATCCGGTAACTATCGTCTTGAGTCCAACCCGGTAAGACACGACTTATCGCCACTGGCAGCAGCCACTGGTTAACAGGATTACCAAAACGAAGGTATGTAAGCC

>3AS4-2

GGATCTAATAGGGCGATTGATTTAGCGGCCGCGAAATTCGCCCTTCATCATCGTGGACCGGTTCCTCCACGAAAAAGACGACCAACAAGATTTCATGGCCGCCATCGCCACCCCGGCTTCCATCTCCGCCGCCGCTGTCGCCGCCACAACTCGGTAAAGCTGCTGGGGCTCATTAAATGTACGGTAGAAATGACACGTGGGATGAGGACAAAATGATCATTCGATAGAGACCTCACAAAACACCAAGGGCGAATTCGTTTAAACCTGCAGGACTAGTACCTTTAGTGAGGGTTAATTCTGAGCTTGGCGTAATCATGGTCATAGCTGTTTCCTGTGTGAAATTGTTATCCGCTCACAATTCCACACAACATACGAGCCGGAAGCATAAAGTGTAAAGCCTGGGGTGCCTAATGAGTGAGCTAACTCACATTAATTGCGTTGCGCTCACTGCCCGCTTTCCAGTCGGGAAACCTGTCGTGCCAGCTGCATTAATGAATCGGCCAACGCGCGGGGAGAGGCGGTTTGCGTATTGGGCGCTCTTCCGCTTCCTCGCTCACTGACTCGCTGCGCTCGGTCGTTCGGCTGCGGCGAGCGGTATCAGCTCACTCAAAGGCGGTAATACGGTTATCCACAGAATCAGGGGATAACGCAGGAAAGAACATGTGAGCAAAAGGCCAGCAAAAGGCCAGGAACCGTAAAAAGGCCGCGTTGCTGGCGTTTTTCCATAGGCTCCGCCCCCCTGACGAGCATCACAAAAATCGACGCTCAAGTCAGAGGTGGCGAAACCCGACAGGACTATAAAGATACCAGGCGTTTCCCCCTGGAAGCTCCCTCGTGCGCTCTCCTGTTCCGACCCTGCCGCTTACCGGATACCTGTCCGCCTTTCTCCCTTCGGGAAGCGTGGCGCTTTCTCATAGCTCACGCTGTAGGTATCTCAGTTCGGTGTAGGTCGTTCGCTCCAAGCTGGGCTGTGTGCACGAACCCCCCGTTCAGCCCGACCGCTGCGCCTTATCCGGTAACTATCGTCTTGAGTCCAACCCGGTAAGACACGACTTATCGCCACTGGCAGCAGCCACTGGTAACAGGATTACCAGACCGAG

>3AS4-3

NAATCTATAGGGCGATTGATTTAGCGGCCGCGAATTCGCCCTTCATCATCGTGGACCGGTTCCTCCACGAAAAAGACGACCAACAAGATTTCATGGCCGCCATCGCCACCCCGGCTTCCATCTCCGCCGCCGCTGTCGCCGCCACAACTCGGTAAAGCTGCTGGGGCTCATTAAATGTACGGTAGAAATGACACGTGGGATGAGGACAAAATGATCATTCGATAGAGACCTCACAAAACACCAAGGGCGAATTCGTTTAAACCTGCAGGACTAGTACCTTTAGTGAGGGTTAATTCTGAGCTTGGCGTAATCATGGTCATAGCTGTTTCCTGTGTGAAATTGTTATCCGCTCACAATTCCACACAACATACGAGCCGGAAGCATAAAGTGTAAAGCCTGGGGTGCCTAATGAGTGAGCTAACTCACATTAATTGCGTTGCGCTCACTGCCCGCTTTCCAGTCGGGAAACCTGTCGTGCCAGCTGCATTAATGAATCGGCCAACGCGCGGGGAGAGGCGGTTTGCGTATTGGGCGCTCTTCCGCTTCCTCGCTCACTGACTCGCTGCGCTCGGTCGTTCGGCTGCGGCGAGCGGTATCAGCTCACTCAAAGGCGGTAATACGGTTATCCACAGAATCAGGGGATAACGCAGGAAAGAACATGTGAGCAAAAGGCCAGCAAAAGGCCAGGAACCGTAAAAAGGCCGCGTTGCTGGCGTTTTTCCATAGGCTCCGCCCCCCTGACGAGCATCACAAAAATCGACGCTCAAGTCAGAGGTGGCGAAACCCGACAGGACTATAAAGATACCAGGCGTTTCCCCCTGGAAGCTCCCTCGTGCGCTCTCCTGTTCCGACCCTGCCGCTTACCGGATACCTGTCCGCCTTTCTCCCTTCGGGAAGCGTGGCGCTTTCTCATAGCTCACGCTGTAGGTATCTCAGTTCGGTGTAGGTCGTTCGCTCCAAGCTGGGCTGTGTGCACGAACCCCCCGTTCAGCCCGACCGCTGCGCCTTATCCGGTAACTATCGTCTTGAGTCCAACCCGGTAAGACACGACTTATCGCCACTGGCAGCAGCCACTGGTAACAGGATTACCAGAGCGAGGTATGTAGGCGGTGCTACAGAGT

>3AS4-4

AATCTAATAGGGCGATTGATTTAGCGGCCGCGAATTCGCCCTTCATCATCGTGGACCGGTTCCTCCACGAAAAAGACGACCAACAAGATTTCATGGCCGCCATCGCCACCCCGGCTTCCATCTCCGCCGCCGCTGTCGCCGCCACAACTCGGTAAAGCTGCTGGGGCTCATTAAATGTACGGTAGAAATGACACGTGGGATGAGGACAAAATGATCATTCGATAGAGACCTCACAAAACACCAAGGGCGAATTCGTTTAAACCTGCAGGACTAGTACCTTTAGTGAGGGTTAATTCTGAGCTTGGCGTAATCATGGTCATAGCTGTTTCCTGTGTGAAATTGTTATCCGCTCACAATTCCACACAACATACGAGCCGGAAGCATAAAGTGTAAAGCCTGGGGTGCCTAATGAGTGAGCTAACTCACATTAATTGCGTTGCGCTCACTGCCCGCTTTCCAGTCGGGAAACCTGTCGTGCCAGCTGCATTAATGAATCGGCCAACGCGCGGGGAGAGGCGGTTTGCGTATTGGGCGCTCTTCCGCTTCCTCGCTCACTGACTCGCTGCGCTCGGTCGTTCGGCTGCGGCGAGCGGTATCAGCTCACTCAAAGGCGGTAATACGGTTATCCACAGAATCAGGGGATAACGCAGGAAAGAACATGTGAGCAAAAGGCCAGCAAAAGGCCAGGAACCGTAAAAAGGCCGCGTTGCTGGCGTTTTTCCATAGGCTCCGCCCCCCTGACGAGCATCACAAAAATCGACGCTCAAGTCAGAGGTGGCGAAACCCGACAGGACTATAAAGATACCAGGCGTTTCCCCCTGGAAGCTCCCTCGTGCGCTCTCCTGTTCCGACCCTGCCGCTTACCGGATACCTGTCCGCCTTTCTCCCTTCGGGAAGCGTGGCGCTTTCTCATAGCTCACGCTGTAGGTATCTCAGTTCGGTGTAGGTCGTTCGCTCCAAGCTGGGCTGTGTGCACGAACCCCCCGTTCAGCCCGACCGCTGCGCCTTATCCGGTAACTATCGTCTTGAGTCCAACCCGGTAAGACACGACTTATCGCCACTGGCAGCAGCCACTGGTAACAGGATTAGCAGAACGAGGGATGTAGGCGGTGCTACAGAGTTCTT


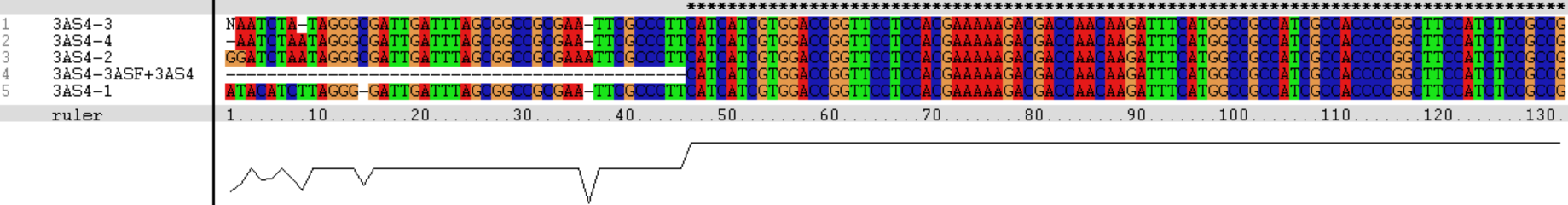

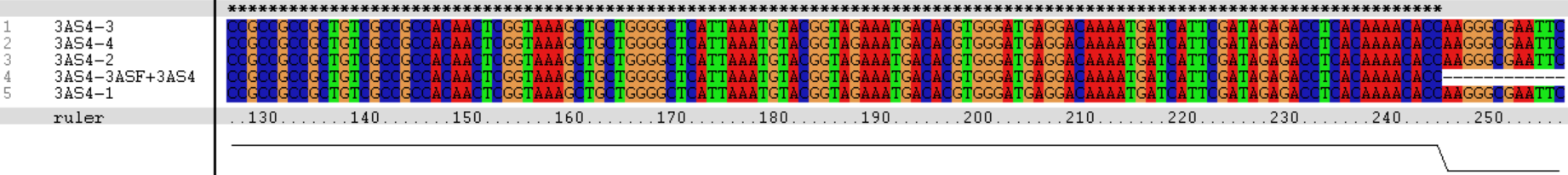

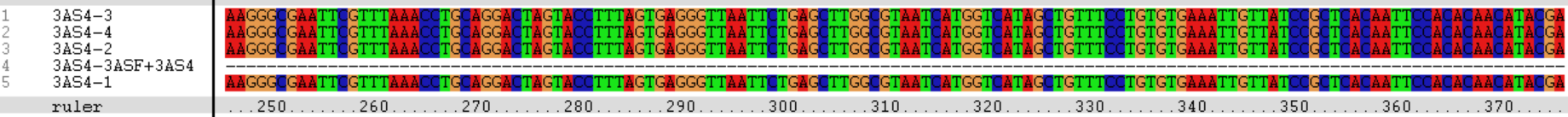

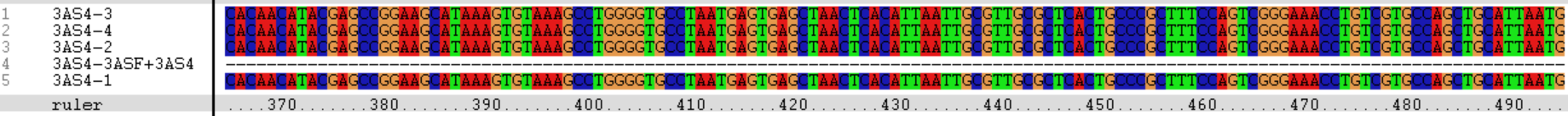

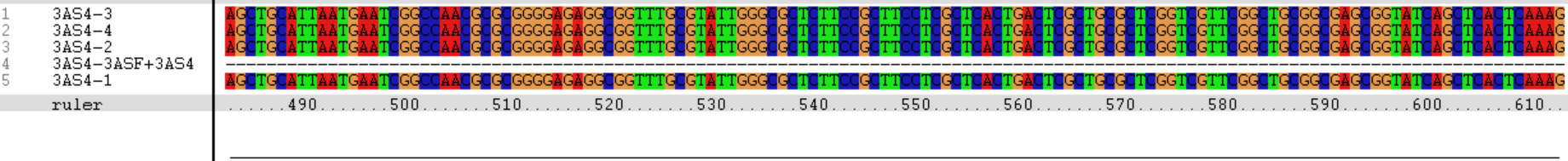

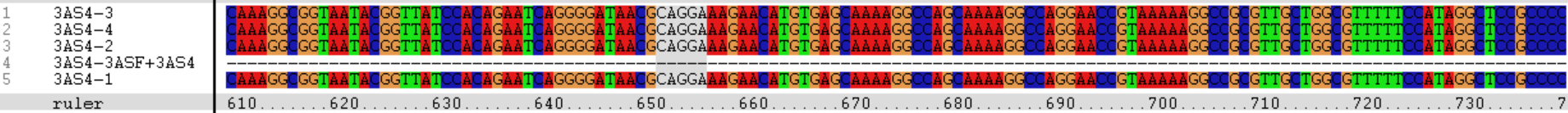

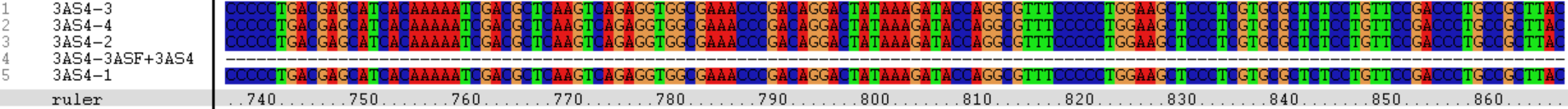

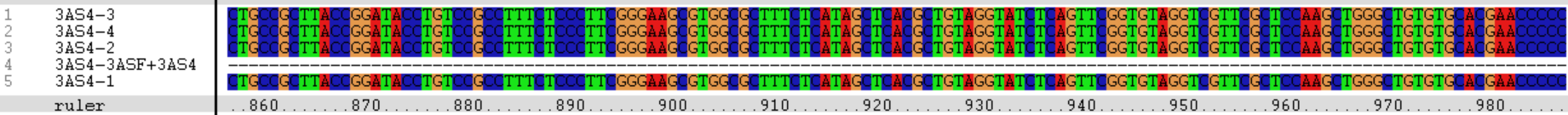

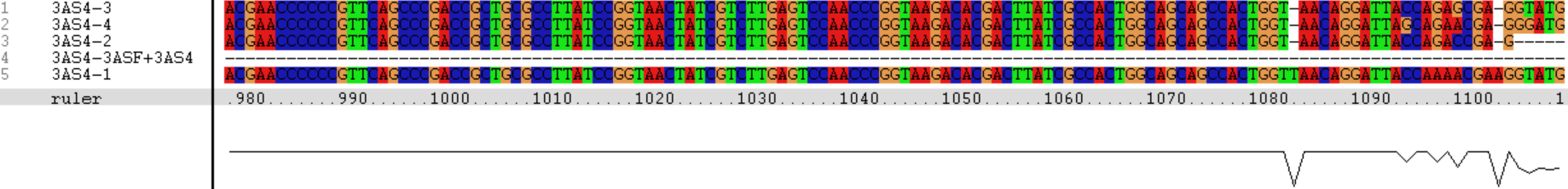

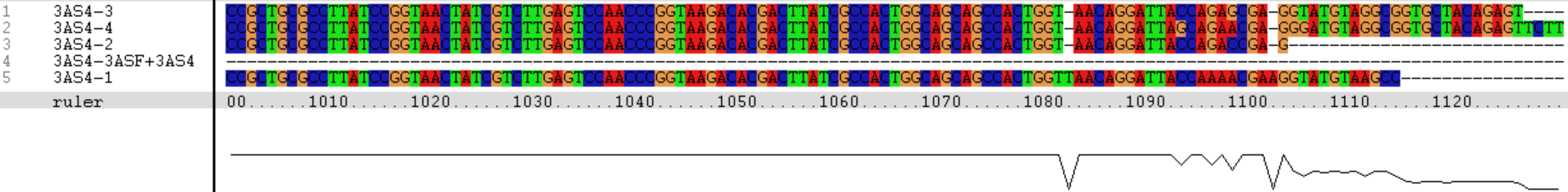


Note: ‘Double underline’ represent primer ‘3ASF+3AS4R’. ‘Yellow highlights’ represent aligned sequences.
